# Endoplasmic reticulum stress-induced histone lactylation mediating immune escape of gastric cancer via PDIA6 overexpression in dendritic cells

**DOI:** 10.1038/s41419-026-08638-9

**Published:** 2026-03-27

**Authors:** Chengshu Tu, Ziqing Wu, Xuxian Zhong, Shengnan Yang, Xi Chen, Simin Li, Youqin Xu, Qiang Zuo

**Affiliations:** 1 Department of Oncology, Nanfang Hospital, Southern Medical University, Guangzhou, China; 2 Department of Pathology, Renmin Hospital of Wuhan University, Wuhan, China; 3 Affiliated Qingyuan Hospital, The Sixth Clinical Medical School, Guangzhou Medical University, Qingyuan People’s Hospital, Guangdong, China; 4 Key Laboratory of Biological Targeting Diagnosis, Therapy and Rehabilitation of Guangdong Higher Education Institutes, The Fifth Affiliated Hospital, Guangzhou Medical University, Guangzhou, China

**Keywords:** Cancer, Immunology, Medical research

## Abstract

Gastric cancer is one of the most prevalent cancers worldwide and is associated with a high mortality rate. Although immunotherapy has achieved some success for many tumor types, the limited response of gastric cancer to immunotherapy poses a challenge. Lactylation is a recently proposed post-translational modification derived from lactate that plays a key role in many physiological processes. In this study, we found that endoplasmic reticulum stress (ERS)-induced histone lactylation attenuated the immune response of dendritic cells (DC), decreased the ability of T cells to kill tumor cells, and enhanced tumor growth. Interestingly, ERS-induced H4K12 lactylation promoted the expression of PDIA6 in DC, leading to weakened immune activity of DC and decreased anti-tumor ability of T cells, thereby promoting gastric cancer immune evasion. Collectively, our work provides new insights into how ERS-induced lactylation modification attenuates the stability of DC to drive immune escape in gastric cancer and provides a promising biomarker for the efficacy of immunotherapy in gastric cancer.

## Introduction

Gastric cancer is the fifth most common cancer and the fourth most common cause of cancer-related deaths worldwide. According to statistics, there are more than 1 million new cases and nearly 800,000 deaths each year [1–3]. The early symptoms of gastric cancer are usually not obvious, and the main features are upper abdominal pain, indigestion, and weight loss [4]. Most gastric cancers are already in an advanced stage when discovered, even with surgery, there is a risk of recurrence, and the side effects of radiotherapy and chemotherapy are relatively severe. Consequently, new treatment methods are being introduced. In recent years, with an increase in research on the tumor microenvironment, some monoclonal antibodies targeting immune escape targets have been approved for immunotherapy of unresectable or metastatic gastric cancer, such as PD-1 inhibitors [5]. PD-1 inhibitors enhance the proliferation and differentiation of T cells in animal models, increase the killing of tumor cells, and reshape the tumor microenvironment [6]. Therefore, focusing on the microenvironment of gastric tumors may provide a reference for immunotherapy in gastric cancer.

The internal environment in which tumor cells are produced and live is known as the tumor microenvironment. In addition to tumor cells, there are fibroblasts, immune cells, inflammatory cells, and glial cells. There are also non-cellular components, including interstitial cells and capillaries, and free biological molecules [7]. The tumor microenvironment has three main characteristics: hypoxia, chronic inflammation, and immunosuppression [8]. Recent studies have shown that hypoxia can reduce the normal folding of proteins in the endoplasmic reticulum, resulting in the accumulation of a large number of unfolded or misfolded proteins. This is known as endoplasmic reticulum (ERS) stress. To alleviate ERS, cells respond with a series of actions to enhance correct protein folding. This response is known as the unfolded protein response (UPR) [9]. However, cancer cells promote ERS to maintain the UPR and meet protein demands during tumor growth [10]. In particular, the endoplasmic reticulum molecular chaperone, glucose-regulated protein 78 (GRP78), is overexpressed in tumors and can be used as a tumor growth marker to promote tumor survival, proliferation, and metastasis [11]. In the tumor microenvironment, immune cells also play an important role [12–14]. Among them, dendritic cells (DC), the most powerful antigen-presenting cells known till date, are worthy of attention in anti-tumor research. Owing to its immunoregulatory ability, it is currently at the center of the immune system. It can capture information from the surrounding environment and transmit it to the appropriate immune cells. Usually, the major histocompatibility complex (MHC) binds to antigens and presents them to T cells in the form of peptide-MHC complexes to stimulate immune responses [15, 16]. However, long-term development of the tumor impairs the maturation of DC and inhibits the antigen presentation effect, thereby preventing the activation of T cells [17, 18]. Currently, the mechanism by which ERS affects the immune function of DC in gastric cancer remains unclear.

In the 1920s, Warburg et al. found that tumor cells preferentially obtain energy through glycolysis in aerobic environments, a phenomenon known as the Warburg effect [19]. Lactate produced from glycolysis by tumors can exert immunosuppressive effects through various mechanisms, especially playing an important role in shaping the function of immune cells [20]. In 2019, Yingming et al. proposed modifications for protein lactylation. They found that lactate produced during metabolism could lead to lactylation modification of histone lysine, thereby affecting the homeostatic regulation of macrophages, inflammatory responses, and tumor-related diseases [21]. This new type of modification provides a deeper and broader approach to lactate research. However, the role of lactylation in immune function of DC has not yet been clarified and it would be interesting to explore the underlying mechanism.

In this study, we demonstrated that ERS plays a key role in promoting the growth of gastric cancer by inhibiting the immune function of DC. Mass spectrometric analysis of histone lactylation revealed that lysine lactylation occurred at position 12. Further mechanistic studies showed that ERS causes H4K12 lactylation of PDIA6 in DC by promoting lactate secretion from gastric cancer cells, thereby inhibiting the immune function of DC. Collectively, we evaluated the mechanistic connection between ERS, lactylation, and DC-mediated immune escape in gastric cancer and identified potential targets for gastric cancer immunotherapy.

## Materials and methods

### Cell culture

Bone marrow-derived dendritic cells (BMDCs) were obtained from 7-week-old SPF C57/BL6 mice. The mice were sacrificed under anesthesia; their femurs and tibias were removed and washed with phosphate-buffered saline (PBS) to obtain a cell suspension. Red blood cells were lysed with RBC lysis buffer for 3 min, and the cell suspension was centrifuged to collect BM cells. BM cells were seeded in 24-well plates and 1 ml of cell culture medium, then 20 ng/ml GM-CSF and 10 ng/ml IL-4 were added. Cells were incubated at 37 °C in a 5% CO_2_ incubator. The medium was changed and cytokines were added on day 3 and 5 to induce the formation of BMDCs. On day 7, GM-CSF, IL-4, and LPS were added to induce maturation of BMDCs [22].

T cells were obtained from 7-week-old SPF C57/BL6 mice. The mice were sacrificed under anesthesia and their spleens were removed and washed with PBS to obtain a cell suspension. CD8^+^ T cells were sorted using lymphocyte separation buffer and a CD8^+^ T Cell Isolation Kit. Tumor-associated macrophages (TAMs) were induced by THP-1 with 100 ng/ml Phorbol-12-myristate-13-acetate (PMA), then 20 ng/ml GM-CSF and 20 ng/ml IL-4 were added.

Mouse gastric cancer cells (MFC), human gastric cancer cells (BGC-823 and AGS) and cancer-associated fibroblasts (CAFs) were derived from ATCC and recently authenticated. They were cultured in 1640 medium (Solarbio, China) containing 10% fetal bovine serum (FBS) at 37 °C and 5% CO_2_. Mouse BMDCs and CD8^+^ T cells were obtained as described above. Cell supernatant was collected after treatment of MFC with Tunicamycin (TM, 5 μg/μL) or after exposure to hypoxic condition, and 10 μmol lactate inhibitor, GSK2837808A (Selleck, USA). The supernatant was used to treat BMDCs, which were subsequently co-cultured with CD8^+^ T cells.

### RNA extraction and RT-qPCR assay

Total RNA was isolated from tumors and cells using the Trizol reagent (Life Technologies, USA). Reverse transcription and RT-qPCR were performed using the PrimeScript RT Reagent Kit (Vazyme, China) according to the manufacturer’s instructions. The primer sequences used for RT-qPCR are listed in Table 1. The experimental data were normalized to the endogenous housekeeping gene β-actin using the 2^−ΔΔCt^ method.

**Table 1:** Primers, sequences, and RT-qPCR conditions for mouse genes.

Genes	Forward primer	Reverse primer
GRP78	CCAAAGATTCAGCAACTGGTG	ATCAAGCAGTACCAGATCACC
CD86	TCAATGGGACTGCATATCTGCC	GCCAAAATACTACCAGCTCACT
CD40	GCAGTGTGTTACGTGCAGTG	TGTGCAGTGGCTTGTCAGTC
MHCII	TGGCCTTTTCATCCGTCACA	ACTGGCAGTCAGGAATTCGG
IL-2	TCACCCTTGCTAATCACTCC	CTGTAGAGCTTGAAGTGGAGC
IFN-γ	CTTTGGACCCTCTGACTTGAG	TCAATGACTGTGCCGTGG
HERPUD1	ACCCCAACAATAACCTCCAG	CCATGCTGTGCTCATAAACG
CRELD2	GAAAACTGCTACAATGTTCCGG	TGCCCGTCACAAATCCTC
PDIA6	CTTTCCTACCATCACTCCCAG	CTCACAACTCATCCTTCTCCAG
CALR	GAGGACTGGGATGAAGAGATG	AGGGTTGTCAATTTCTGGGTG
β-actin	TCAAGATCATTGCTCCTCCTGAG	ACATCTGCTGGAAGGTGGACA

*GRP78* glucose regulated protein78kD, *IL-2* interleukin 2, *IFN-γ* interferon-gamma, *HERPUD1* homocysteine-inducible, endoplasmic reticulum stress-inducible, ubiquitin-like domain member 1, *CRELD2* Cysteine-Rich with EGF-Like Domain Protein 2, *PDIA6* protein disulfide isomerase family A, member 6.

### Enzyme-linked immunosorbent assay

After processing the cells, the cell supernatant was collected and the level of IL-12 and Granzyme B were measured using ELISA. ELISA kits (Cusabio) were used according to the manufacturer’s instructions.

### Western blot analysis

Cells were lysed on ice using RIPA lysis buffer, and centrifuged at 15,000 *g* for 30 min at 4 °C. The supernatant was collected, and the protein concentration was measured using a BCA assay kit. Proteins were separated using SDS-PAGE and transferred onto PVDF membranes (Millipore Corporation, USA). The samples were incubated overnight at 4 °C with the specific primary rabbit anti-PDIA6 antibody (Proteintech, China) at a dilution of 1:3000, mouse-GRP78 antibody (Proteintech, China) at a dilution of 1:2000, rabbit anti-Pan-kla antibody (PTM-BIO, China) at a dilution of 1:1000, rabbit anti-H4K12 antibody (PTM-BIO, China) at a dilution of 1:1000, and mouse anti-GAPDH antibody (Ray Monoclonal Antibody, Beijing, China) at a dilution of 1:5000. The membrane was then incubated with the secondary antibody for 1 h. Specific protein bands were visualized using an ECL Detection Kit (Vazyme, China) and analyzed using ImageJ software.

The cells were collected for protein extraction; 50 μl of protein A/G beads were incubated at 4 °C on the rotor overnight with the corresponding concentration of PDIA6 (Proteintech, China) or H4K12 (PTM-BIO, China) antibodies and centrifuged at 12,000 *g* for 1 min. The protein A/G beads were washed for three times, and the proteins were analyzed.

### RNA knockdown, lentiviral infection, and plasmid transfection

The siRNA targeting PDIA6 was provided by RIB-BIO (China), and Lipofectamine^TM^ 3000 Reagent (Invitrogen, USA) was used to promote transfection. All siRNA target sequences are summarized in Table 2. Cells were seeded in 6-well plates for lentivirus infection. Lentiviruses containing H4K12 WT or H4K12R mutants were obtained from Shanghai Genechem Co., Ltd. The lentiviruses were added to the cells according to the manufacturer’s instructions, and the culture medium was changed after 24 h. One week after the addition of polybrene, the cells were used for further experiments.

**Table 2:** Targeted sequences of siRNAs.

Genes	Targeted sequences
si-m-Pdia6_001	CCCTACAATCAAGATATTT
si-m-Pdia6_002	ACGAAGACATAGCCAAGAA
si-m-Pdia6_003	GCTGACAAGTACAAGAAGA

### Identification of lactylation sites by LS-MS/MS

First, cell histones were extracted using a histone extraction kit (Solarbio, China), followed by the preparation of protein samples. Protein samples were then isolated by SDS-PAGE and stained with Richfast SDS-PAGE Staining Buffer (Prosperich, China). The colloids were cut for MS analysis to determine the location of the lactylation group. The MS assay was performed by Shenzhen Wininnovate Biotechnology.

### Transcriptome sequencing and analysis

After processing, the cells were collected and Trizol reagent was added. The host company used the platform for transcriptome analysis to complete the gene expression and differential expression analyses.

### Establishment and treatment of a subcutaneous tumor model with ERS response in mice

Male C57/BL6 mice (age 5–6 weeks, *n* = 3) were purchased from the Southern Medical University Laboratory Animal Management Center and randomly divided into two groups. All procedures involving experimental animals were performed in accordance with the protocol approved by the Nanfang Hospital Animal Ethics Committee. 5 × 10^6^ MFC cells were injected subcutaneously into the flank of each mouse, and the mice were intraperitoneally injected with DMSO or TM (0.35 μg/g, twice a week). When the tumors became palpable, the ERS-responsive tumor mice were randomly divided into two groups. siRNA or lactate inhibitors were intratumorally injected into ERS-responsive tumor mice. After 7 days of tumor growth in mice, the diameter and width of the tumors were measured twice a week and used to calculate the tumor volumes using the formula V = 0.5 × D × W^2^ (V, volume; D, diameter; and W, width). All mice were euthanized at appropriate time points. The tumors were removed and photographs were taken.

### Flow cytometry

Cell counts were determined using flow cytometry. All the antibodies were obtained from BioLegend (USA). All data were analyzed using the FlowJo software. Cells were centrifuged, and the cell pellet was resuspended in 100 μl PBS. Next, the cell suspension was incubated with anti-mouse CD45-PE-Cy7, anti-mouse CD11c-FITC, anti-mouse CD86-PerCP, MHCII-BV421, CD8-BV510, and IFN-γ-PE for 20 minutes. Finally, the flow cytometry results were analyzed.

### Immunofluorescence and immunohistochemistry staining

Paraffin sections of the tumor were deparaffinized in water, and antigen retrieval was performed at room temperature on slides using a citric acid retrieval solution. The slides were then blocked with bovine serum albumin (BSA) for 30 min. The slides were incubated overnight with 1:200 anti-GRP78 antibody (Proteintech, China), 1:200 PDIA6 antibody (Proteintech, China) and 1:2000 CD8 antibody (Proteintech, China) at 4 °C. Subsequently, the slides were incubated with the corresponding secondary antibodies. After washing with PBS, the slides were counterstained with DAB and hematoxylin. Finally, the slides were dehydrated with graded ethanol and the images were examined using an optical microscope.

The slides were washed three times with PBS and blocked with 5% BSA for 30 min. Then the slides were incubated overnight with 1:1000 anti-CD11c antibody (Proteintech, China), 1:100 PDIA6 antibody (Proteintech, China), 1:100 Pan-kla antibody (PTM-BIO, China) and 1:100 H4K12 antibody (PTM-BIO, China) at 4 °C, followed by incubation with the corresponding fluorescent secondary antibody at room temperature in the dark for 1 h, and DAPI staining in the dark for 10 min. Finally, images were captured using a fluorescence microscope.

### Analysis of lactate level

Cell supernatants and tumor tissues were collected and washed with pre-chilled PBS. Cell supernatant was removed after centrifugation and lactate reagent buffer was added. Tissue was centrifuged at 12,000 *g* for 5 min at 4 °C, and the supernatant was transferred to a new tube. Supernatant was collected for the determination of lactate level using CheKine™ Lactate Assay Kit (Abbkine, China).

### Bioinformatics

The public database GEPIA (http://gepia.cancer-pku.cn/) was explored to assess the expression of GRP78 in the tissues of gastric cancer to examine the potential relationship between GRP78 and PDIA6. The relationship between PDIA6 expression and the overall survival of gastric cancer patients was analyzed.

### Statistical analysis

All data were presented as mean ± standard deviation (S.D.) using GraphPad prism 10. The statistical significance of the differences was compared using Student’s *t* test. Three or more groups were compared using a one-way ANOVA. *P* value < 0.05 was considered to be statistically significant.

## Results

### ERS is essential for the progression of gastric cancer

**Fig. 1:**
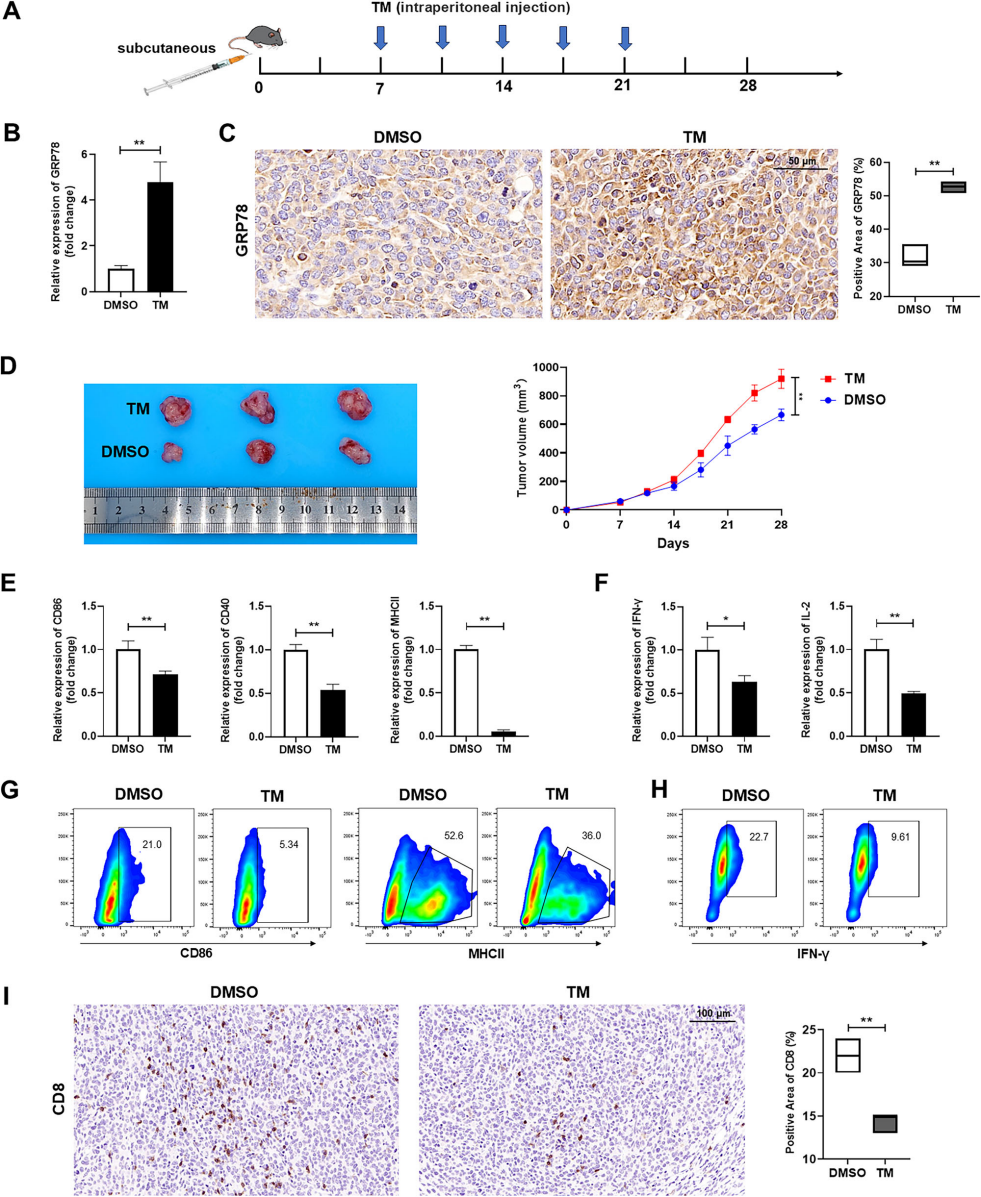
ERS promotes the progression of gastric cancer. **A** Establishment of mice model of ERS-status gastric cancer: Mice were subcutaneously injected with MFC on day 0, intraperitoneally injected with TM (0.35 μg/g) on day 7, twice a week. These mice were sacrificed 28 days later. **B** RT-qPCR was used to detect the RNA expression level of GRP78 in subcutaneous tumors. **C** The protein expression level of GRP78 was detected by immunohistochemistry. **D** The subcutaneous tumor was taken and photographed, and the growth curve was drawn according to the volume of the tumor using the formula V = 0.5 × D × W^2^, with a unit of mm^3^. **E**, **F** RT-qPCR was used to detect RNA expression level of CD86, CD40, MHCII, IFN-γ, and IL-2 in mice. **G**, **H** The tumor suspension was prepared and the proportion of CD86, MHCII, and IFN-γ in the tumor was detected using flow cytometry. **I** Immunohistochemistry was used to detect the distribution of CD8^+^ T cells. *N* = 3, data expressed as Mean ± SD for different groups. *P < 0.05, **P < 0.01.

To explore the effects of ERS on the development of gastric cancer, we established a mouse model. MFC cells were subcutaneously injected into the mice. Tumors formed approximately 7 days later, at which time TM was injected intraperitoneally into the mice twice a week. After 28 days, the mice were anesthetized and sacrificed, then the tumors were removed (Fig. 1A). GRP78, a molecular marker of ERS, is highly expressed in ERS and plays an important role in promoting the formation and development of tumors [10]. To validate the ERS state of mouse subcutaneous tumor models, we examined the RNA (Fig. 1B) and protein expression level (Fig. 1C) of GRP78 in subcutaneous tumor and found that GRP78 expression was significantly elevated in the subcutaneous tumors of mice in the TM group compared with that of those in the DMSO group. The subcutaneous tumor of the mice in the TM group was significantly enlarged, and the tumor growth rate in the TM group was significantly higher than that in the DMSO group (Fig. 1D). These results suggest that the injection of TM into mice subcutaneous tumor models successfully induced an ERS response and significantly promoted tumor growth.

To explore the effect of ERS on immune function in gastric cancer, we extracted the RNA from the subcutaneous tumor of mice and detected the expression level of DC-related markers CD86, CD40 and MHCII, and T cells-related markers, IFN-γ and IL-2. The results showed that the expression levels of these molecular markers were downregulated in the TM group compared with those in the DMSO group (Fig. 1E, F). Flow cytometry results showed a reduction in CD86, MHCII, as well as IFN-γ proportion in the TM group compared to that in the DMSO group (Fig. 1G, H). We also examined the distribution of CD8^+^ T cells in subcutaneous tumors using immunohistochemistry and found that the distribution of CD8^+^ T cells was significantly reduced in the TM group (Fig. 1I). These results suggest that ERS plays an important role in inhibiting the maturation and activation of DC and decreasing the activation of T cells.

### ERS inhibits the immune function of DC in the gastric cancer microenvironment in vitro

To further explore the effect of ERS on DC immune function in gastric cancer, we first stimulated MFC using TM (5 μg/μL) and hypoxia (1% O_2_) according to the literature and previous experimental conclusions of our group [23]. The model of MFC in ERS state was constructed in vitro to simulate the tumor ERS microenvironment. To explore the optimal time point, stimulation times of 12 h and 24 h were selected. After stimulation, the normal culture medium was replaced for 24 h and the cell supernatant was collected for subsequent experiments. RNA and protein were extracted to determine the expression levels of GRP78. The results showed that GRP78 expression in MFC increased after exposure to TM or hypoxic stimulation (Fig. S1A, B, C, D). These results suggested that both TM and hypoxic stimulation of MFC can produce ERS responses, leading to the upregulation of GRP78. To investigate the effect of ERS on DC function, we used the classical culture method for BMDCs and obtained BMDCs from mouse bone marrow for stimulation. We examined the secretion level of IL-12 using ELISA, and results showed that the mature DC (mDC) secretion level of IL-12 was significantly increased compared to the immature (iDC) secretion level (Fig. S1E), and the RNA expression showed that level of CD86, CD40, and MHCII was significantly increased in mDC (Fig. S1F). Mature BMDCs were thus selected for further experiments.

**Fig. 2:**
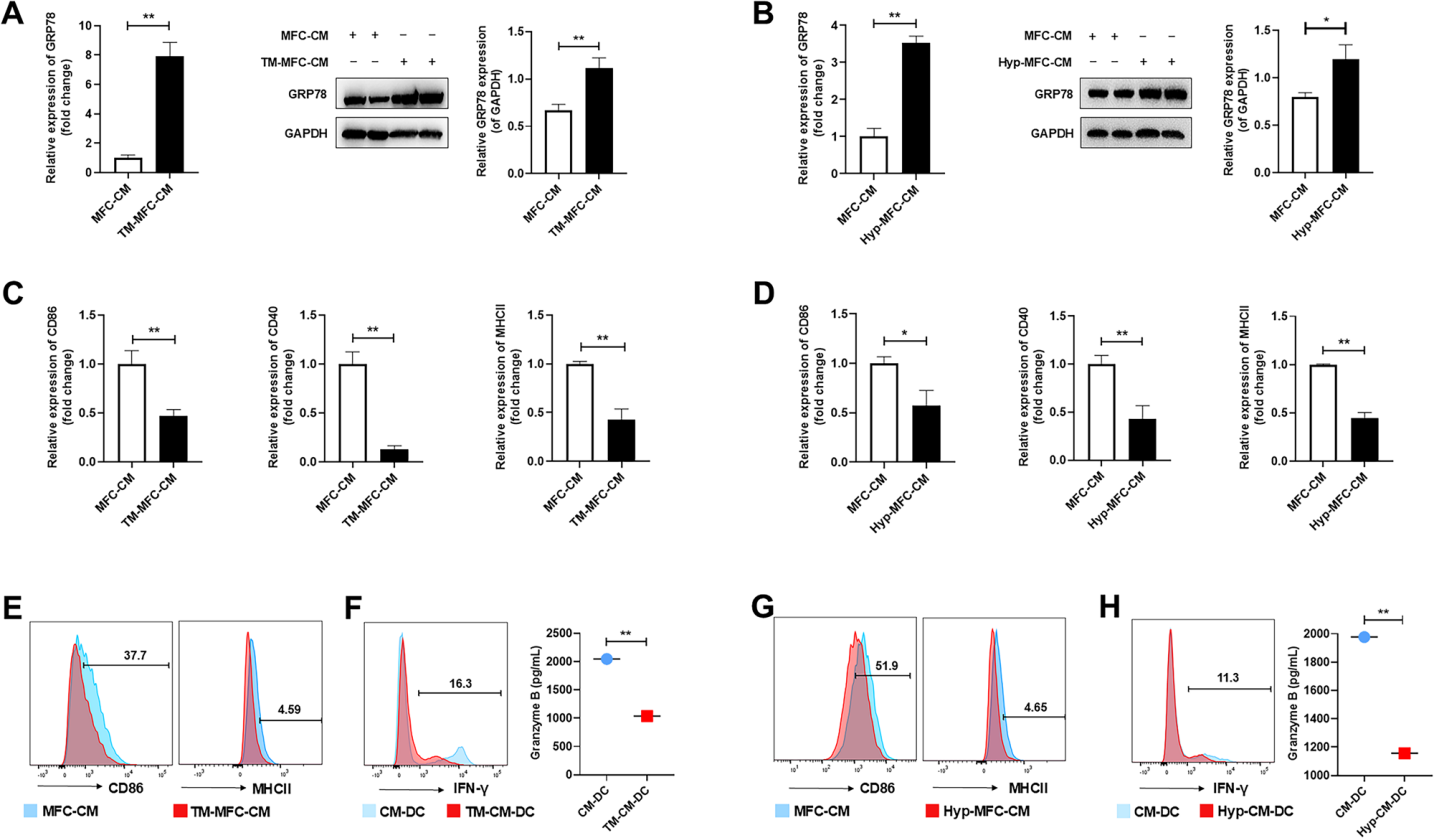
ERS inhibits the immune function of DC. **A**, **B** Supernatants of TM- and hypoxia-stimulated MFC were collected. BMDCs were then treated by these supernatants, and RNA and protein expression level of GRP78 was detected by RT-qPCR and western blotting. **C**, **D** RT-qPCR was used to detect the RNA expression level of CD86, CD40, and MHCII in BMDCs. **E**, **G** Proportion of CD86 and MHCII detected using flow cytometry. **F**, **H** Flow cytometry and ELISA were used to detect the proportion of INF-γ and Granzyme B secretion level in CD8^+^ T cells. *N* = 3, data expressed as Mean ± SD for different groups. *P < 0.05, **P < 0.01.

Next, BMDCs were stimulated using pre-treated supernatants (TM and hypoxia). After 24 h of stimulation, BMDCs were collected and GRP78 expression was detected by RT-qPCR and western blotting. Compared to the common MFC supernatant, both the RNA and protein expression level of GRP78 was elevated after stimulation (Fig. 2A, B). Subsequently, the expression of relevant markers in BMDCs was examined. The results showed that the RNA level of CD86, CD40, and MHCII was decreased in BMDCs after stimulation (Fig. 2C, D). To explore the influence of BMDCs on the ERS state in CD8^+^ T cells, BMDCs were stimulated and co-cultured with CD8^+^ T cells. Flow cytometry and ELISA were used to detect cell activation-associated phenotypic expression. Compared to the common supernatant, the proportion of CD86 and MHC II in BMDCs decreased after stimulation (Fig. 2E, G). Meanwhile, the proportion of INF-γ and Granzyme B secretion were decreased in T cells co-cultured with TM/Hypoxia-CM-DC compared to that with CM-DC (Fig. 2F, H). These results suggest that ERS inhibits the immune function of DC by downregulating the expression of the mature DC phenotype and inhibiting the activation of CD8^+^ T cells, thereby promoting immune escape in gastric cancer.

### ERS promotes the growth of gastric cancer by increasing the lactate level

ERS can cause various changes in cell metabolic functions, including inhibiting the synthesis of amino acids, increasing the synthesis of fatty acids, and altering glycolysis [24–26]. To explore the effect of ERS on the product of glycolysis, lactate, in the gastric cancer microenvironment, the lactate level was measured. Results showed that the lactate level in the subcutaneous tumors of mice in the TM group was significantly higher than that in the mice in the DMSO group (Fig. S2A). Simultaneously, after TM and hypoxia stimulation, the secretion of lactate in the supernatant from MFC was also increased compared with that in the control group (Fig. S2B). These results showed that ERS promotes the upregulation of lactate derived from gastric cancer cells. Meanwhile, to verify the effect of tumor-associated macrophages (TAMs) or cancer-associated fibroblasts (CAFs) on the secretion of lactate by gastric cancer cells, we used gastric cancer cells: BGC-823 and AGS, which were co-cultured with TAMs and CAFs, respectively. The supernatant was collected for the detection lactate, and it was found that there was no significant difference in the amount of lactate (Fig. S2C). It was concluded that TAMs and CAFs had no distinct effect on the secret of lactate in BGC-823 and AGS.

**Fig. 3:**
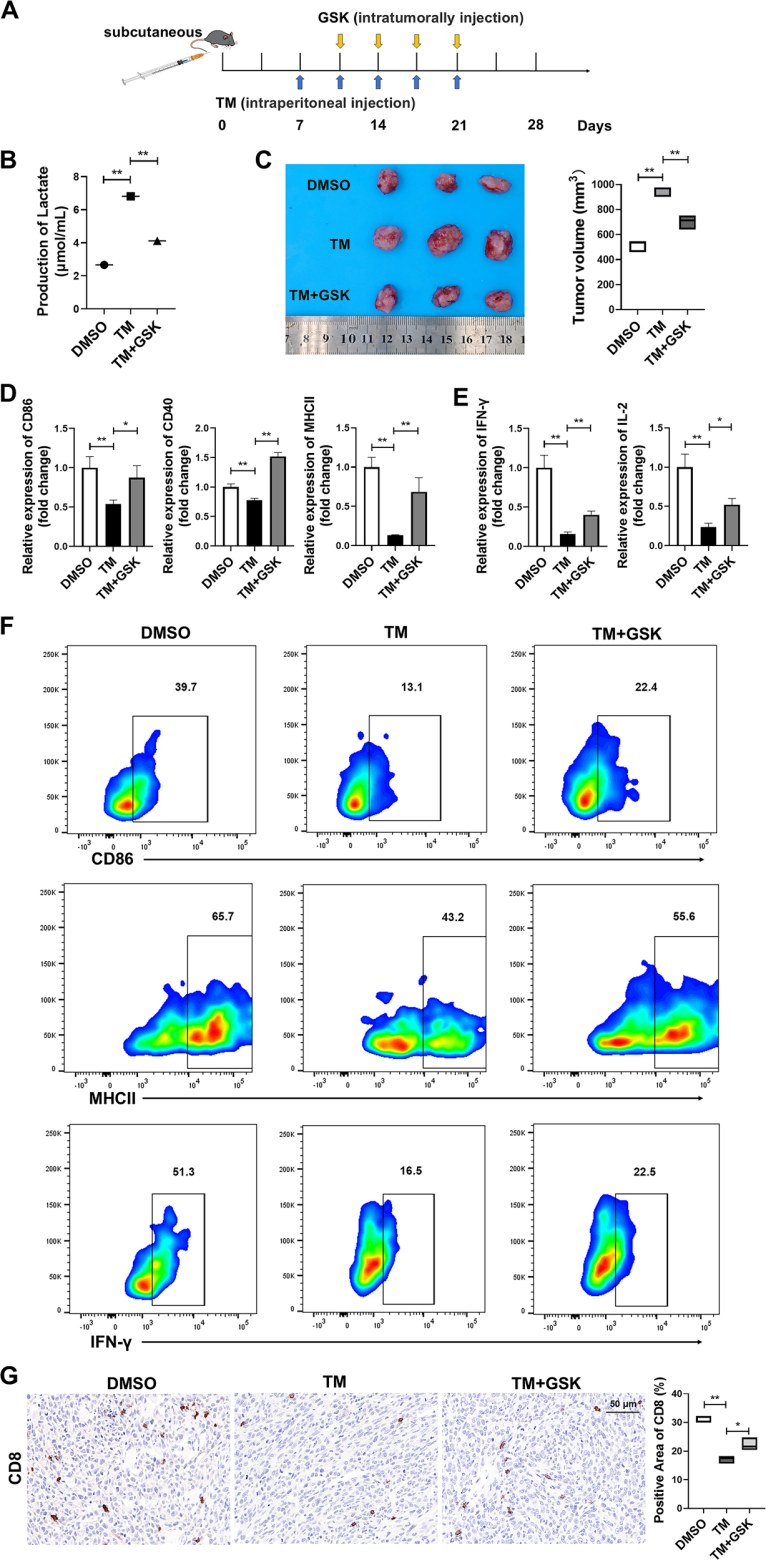
ERS promotes gastric cancer progression by upregulating lactate levels. **A** Subcutaneous tumor mice in ERS status were treated with GSK: mice were subcutaneously injected with MFC on day 0, intraperitoneally injected with TM on day 7, as well as intratumorally injected with GSK a second time, twice a week. After 28 days, the mice were euthanized. **B** The subcutaneous tumor tissue was collected from mice to detect lactate secretion level. **C** The subcutaneous tumor was taken and photographed, and the tumor volume was calculated. **D**, **E** After using GSK, RT-qPCR was used to detect the RNA expression level of CD86, CD40, MHCII, IFN-γ, and IL-2 in mice subcutaneous tumor tissue. **F** Flow cytometry was used to detect the proportion of CD86, MHCII, and IFN-γ. **G** Immunohistochemistry was used to detect of the distribution of CD8^+^ T cells. *N* = 3, data expressed as Mean ± SD for different groups. *P < 0.05, **P < 0.01.

To test whether ERS could promote the growth of gastric cancer by lactate secretion, MFC were subcutaneously injected into mice, which formed approximately 7 days later, at which time TM was intraperitoneally injected. GSK 2837808 A, a lactate dehydrogenase A (LDHA) inhibitor, was injected intratumorally at the same time as the second TM injection to inhibit lactate production. After 28 days. The mice were sacrificed under anesthesia (Fig. 3A). Results showed that the lactate level in the TM group was higher than that in the DMSO group (Fig. 3B), and that the tumor volume in the TM group also increased (Fig. 3C). Compared to the TM group, the lactate level (Fig. 3B) and tumor volume (Fig. 3C) were reduced in the TM + GSK group. These results show that ERS promotes tumor development by increasing lactate level in the tumor microenvironment.

To investigate the effect of ERS-induced lactate increase on immune cells in gastric cancer cells, the expression level of CD86, CD40, MHCII, IFN-γ, and IL-2 in mice subcutaneous tumor tissue was measured. The results showed that the expression level of these molecular markers in the TM group was downregulated compared with that in the DMSO group. While after the addition of GSK, the expression of these co-stimulatory molecules and cytokines was upregulated (Fig. 3D, E). Flow cytometry results showed that the expression change of CD86, MHCII, and IFN-γ showed the same trend (Fig. 3F). The distribution of CD8^+^ T cells in the subcutaneous tumor tissue was detected using immunohistochemistry. Results showed that the distribution of CD8^+^ T cells in the TM group was significantly reduced compared with that in the DMSO group. Whereas after the addition of GSK, the distribution of CD8^+^ T cells increased (Fig. 3G). These results show that ERS reduced the maturation and activation of DC and the killer activity of CD8^+^ T cells by increasing lactate level in gastric cancer, thereby promoting the growth and immune escape of gastric cancer.

### ERS affects DC cell function by upregulating lactate levels in vitro

**Fig. 4:**
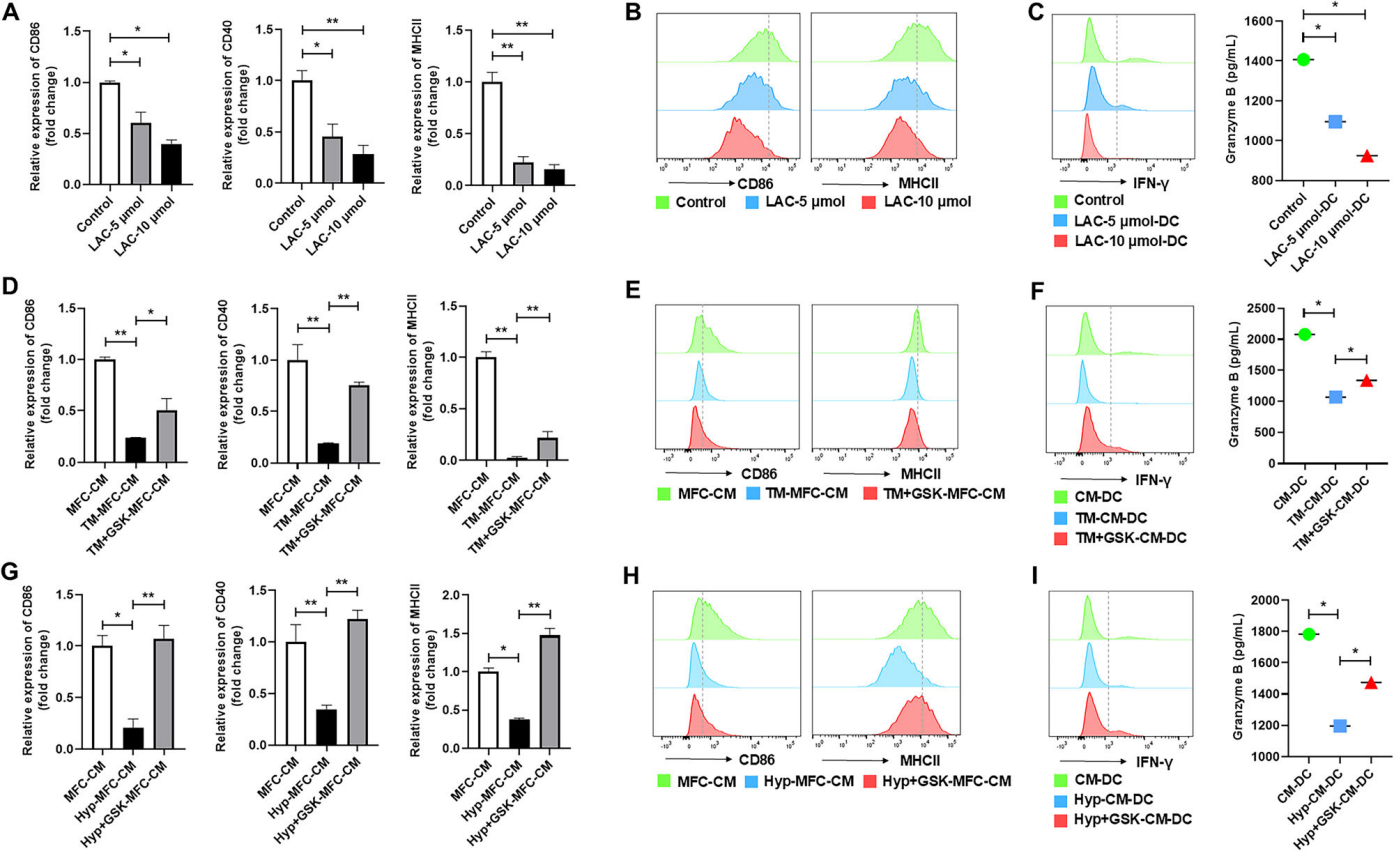
ERS inhibits the immune function of DC by upregulating lactate. **A** Gradient lactate stimulation was performed on BMDCs, and RT-qPCR was used to detect the RNA expression level of CD86, CD40, and MHCII in BMDCs. **B** The proportion of CD86 and MHCII was detected using flow cytometry. **C** BMDCs were co-cultured with CD8^+^ T cells, then the proportion of INF- γ and the secretion level of Granzyme B were detected. **D**, **G** Supernatant was treated with GSK to stimulate BMDCs, and RT-qPCR was used to detect the RNA expression of CD86, CD40, and MHCII in DC. **E**, **H** Flow cytometry was used to detect the proportion of CD86 and MHCII in BMDCs stimulated with the supernatant. **F**, **I** BMDCs were co-cultured with CD8^+^ T cells, then flow cytometry and ELISA were used to detect the proportion of INF-γ and secretion level of Granzyme B. *N* = 3, data expressed as Mean ± SD for different groups. *P < 0.05, **P < 0.01.

First, to explore the effect of lactate on DC function, BMDCs were directly stimulated with different concentrations of lactate (5 μmol, 10 μmol). With the increase in lactate concentration, the RNA expression level of CD86, CD40, and MHCII was gradually decreased (Fig. 4A). The flow cytometry results also showed that the proportion of CD86 and MHCII was gradually decreased (Fig. 4B). Lactate-stimulated BMDCs were co-cultured with CD8^+^ T cells, and the proportion of IFN-γ and the secretion of Granzyme B in T cells were gradually decreased (Fig. 4C). These results indicate that lactate can reduce the production of co-stimulatory molecules in DC, thereby preventing the activation of T cells.

To further verify that ERS could affect DC function by promoting lactate secretion from gastric cancer cells, MFC was treated with GSK and the supernatant was collected to stimulate BMDCs. The results showed that compared with that in the common supernatant, the RNA expression and proportion of CD86, CD40, and MHCII in BMDCs stimulated by the pretreated supernatant were decreased, whereas the RNA expression and proportion of these co-stimulatory molecules were increased after addition of the GSK-stimulated supernatant (Fig. 4D, E, G, H). When the BMDCs were co-cultured with CD8^+^ T cells, it was observed that compared with that in CM-DC, the proportion of INF-γ and the secretion of Granzyme B were decreased in T cells co-cultured with TM/Hyp-DC, while the proportion of INF-γ and secretion of Granzyme B in T cells co-cultured with TM/Hyp + GSK-DC were increased (Fig. 4F, I). The above in vitro experimental results show that ERS inhibits the maturation of DC, prevent the induction of T cell activation and secretion of immune killer factors by promoting the secretion of lactate in tumor cells.

### The increase of lactate induced by ERS plays an important role in regulating histone lactylation at H4K12 in DC

**Fig. 5:**
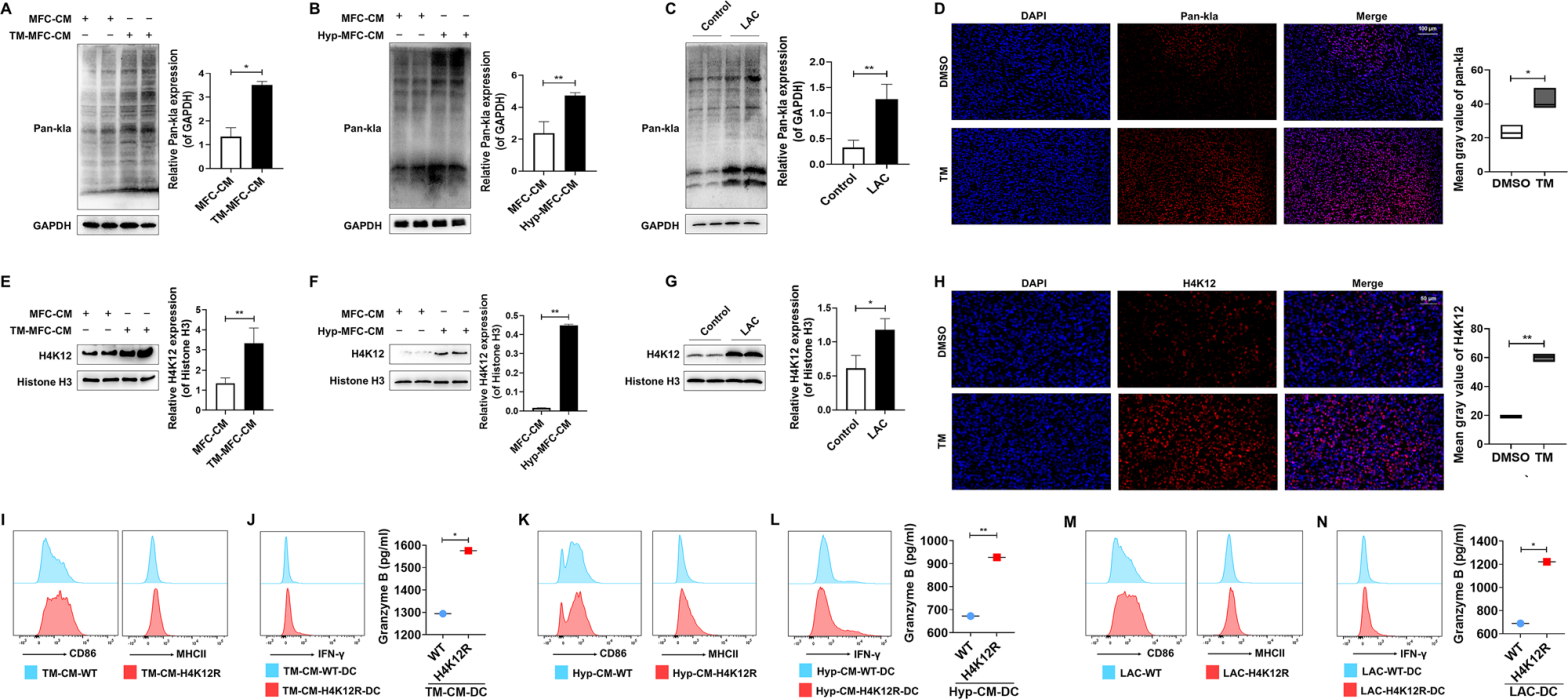
The H4K12 lactylation induced by ERS inhibits the immune function of DC. **A**, **B**, **C** BMDCs were treated with supernatant and lactate, then western blotting was used to detect the expression level of Pan-kla. **D** Immunofluorescence was used to detect the level of Pan-kla in the subcutaneous tumor tissue from mice. **E**, **F**, **G** BMDCs were treated with supernatant and lactate. Nuclear protein was extracted, and the expression level of H4K12 was detected by western blotting. **H** Immunofluorescence was used to detect the level of H4K12 in the subcutaneous tumor tissue of mice. **I**, **K**, **M** Lentivirus was used to infect BMDCs for H4K12R point mutation, then pretreated-supernatant/lactate was used to stimulate BMDCs and associated phenotypes were detected. **J**, **L**, **N** The infected BMDCs were co-cultured with CD8^+^ T cells to detect the related cytokines. *N* = 3, data expressed as Mean ± SD for different groups. *P < 0.05, **P < 0.01.

We validated that ERS can promote lactate secretion and next investigated whether lactate can affect the function of DC through histone lactylation. First, the BMDCs were stimulated using pretreated-supernatant (TM and hypoxia) and lactate, then results showed that Pan-kla expression level was elevated in BMDCs stimulated by pretreated supernatant (TM and hypoxia) compared to that with common supernatant (Fig. 5A, B). Moreover, the expression level of Pan-kla in BMDCs of LAC group was increased compared with that in the control group (Fig. 5C). We also examined level of Pan-kla in subcutaneous tumor tissues of mice by immunofluorescence and found that compared with that in the DMSO group, the level of Pan-kla was significantly increased in the TM group (Fig. 5D).

To identify specific regulatory sites for the lactylation of histone, BMDCs were stimulated with lactate to extract nuclear protein. It was found that the level of Pan-kla was also elevated in the LAC group compared with that in the control group (Fig. S3A). Histone was extracted from BMDCs after lactate stimulation and the histone SDS-PAGE bands were collected for mass spectrometry detection. The secondary mass spectrometry peak map of the histone showed the lactylation of lysine at position 12 (Fig. S3B). We subsequently validated the elevated H4K12 expression in BMDCs stimulated with lactate and supernatant. Results showed that expression level of H4K12 was increased after stimulation (Fig. 5E, F, G). The immunofluorescence results also showed that compared with that in the DMSO group, H4K12 level was elevated in the subcutaneous tumor tissues of mice in the TM group (Fig. 5H).

To further validate the effect of lactylation on DC, BMDCs were infected with a lentivirus for point mutations at the H4K12 site to inhibit H4K12 function, followed by stimulation with the pretreated supernatant (TM/hypoxia) and lactate. These results showed the proportion of both CD86 and MHCII was upregulated in the H4K12R-mutant group compared to that in the WT group (Fig. 5I, K, M). In the co-culture of lentivirus-infected BMDCs with T cells, the proportion of IFN-γ and Granzyme B secretion were upregulated in the H4K12R-mutant group compared to that in the WT group (Fig. 5J, L, N).

### ERS affects the expression of PDIA6 in DC via H4K12 lactylation and results in immunosuppression

**Fig. 6:**
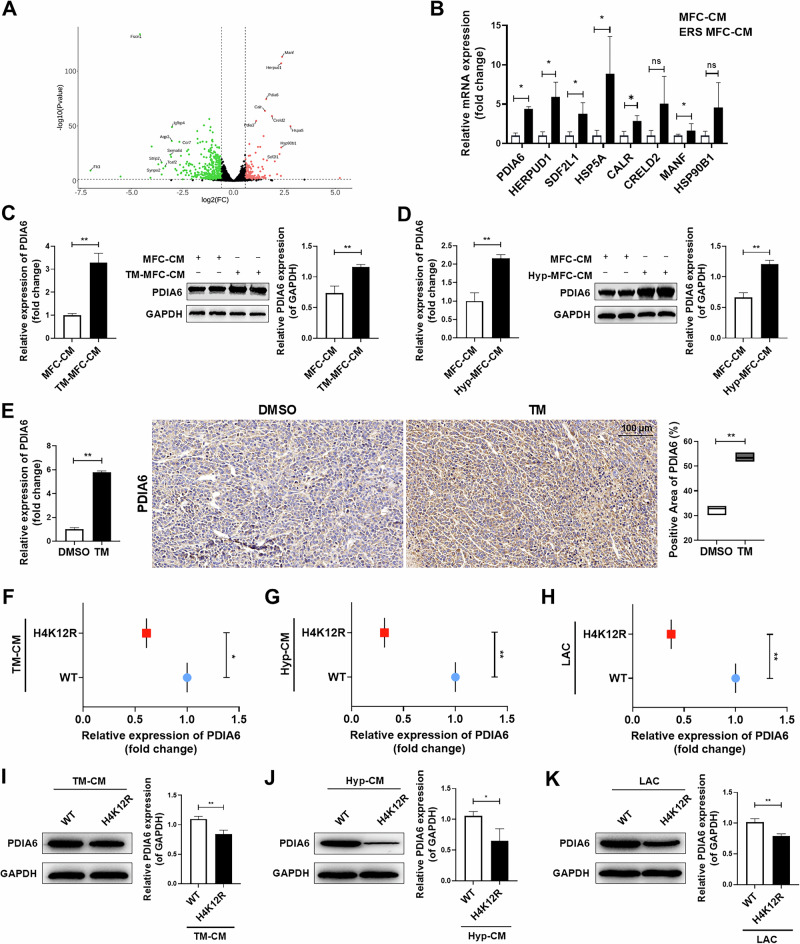
ERS enhanced the expression of PDIA6 via H4K12 lactylation in DC. **A**, **B** After BMDCs were pretreated with the supernatant, transcriptome sequencing was performed to detect gene expression and the results were verified by RT-qPCR. **C**, **D** BMDCs were stimulated by the supernatant, and RNA and protein expression level of PDIA6 was detected by RT-qPCR and western blotting. **E** RT-qPCR was used to detect the RNA expression level of PDIA6 and protein expression level of PDIA6 was detected by immunohistochemistry in subcutaneous tumors. **F**, **G**, **H** Lentivirus was used to infect BMDCs for H4K12R point mutation, then pretreated-supernatant/lactate was used to stimulate BMDCs, and RNA expression of PDIA6 was detected. **I**, **J**, **K** Protein expression of PDIA6 was detected. *N* = 3, data expressed as Mean ± SD for different groups. *P < 0.05, **P < 0.01.

To explore the effect of lactate on the function target gene in DC, we stimulated BMDCs using the pretreated supernatant, followed by transcriptome sequencing. Based on the magnitude of expression differences and a literature search, several immunosuppressive genes, including PDIA6, HERPUD1, SDF2L1, HSP5A, CALR, CRELD2, MANF, and HSP90B1, were selected to verify whether their expression was consistent with the sequencing results (Fig. 6A, B). We observed that the expression of PDIA6 was relatively significant and stable, and it was also related to ERS-related gene. Consequently, we selected to further the related changes of PDIA6. After stimulation, PDIA6 expression in BMDCs was detected by RT-qPCR and western blotting. Compared to that in the common supernatant, both RNA and protein expression level of PDIA6 was elevated after stimulation (TM and hypoxia) (Fig. 6C, D). Compared to that in the DMSO group, the RNA and protein expression level of PDIA6 in the subcutaneous tumor was also elevated in the TM group (Fig. 6E). Moreover, we used lactate to directly stimulate BMDCs and found that the RNA and protein expression level of PDIA6 was up-regulated with increased lactate concentration (Fig. S4A). After adding GSK, the RNA and protein expression level of PDIA6 was reduced compared to ERS (TM/Hypoxia)-stimulated BMDCs (Fig. S4B, C). The in vivo results also showed a trend of change in TM + GSK group compared with that in the TM group. (Fig. S4D). More importantly, consistent decrease was found about the expression of PDIA6 and H4K12 in TM + GSK group compared with that in the TM group. (Fig. S4I). To further verify the effect of H4K12 lactate on PDIA6 in DC, we used a CO-IP experiment to identify whether H4K12 and PDIA6 interact directly (Fig. S4E). Besides this, BMDCs were infected with lentivirus for point mutations at the H4K12 site to inhibit H4K12 function, followed by stimulation of infected BMDCs with the pretreated supernatant (TM/hypoxia) and lactate. These results showed the decrease in RNA and protein expression level of PDIA6 after H4K12 function was inhibited (Fig. 6F, G, H, I, J, K). To prove the direct relationship between H4K12 lactylation and PDIA6, we performed ChIP-qPCR on the promoter of PDIA6 with H4K12. We found that H4K12R mutation would lead to impaired binding to PDIA6 promoters, suggesting that PDIA6 was regulated by the level of H4K12 lactylation (Fig. S4F, G, H).

To further verify that ERS affects DC function by promoting the expression of PDIA6, we added the supernatant (TM and hypoxia) after treatment with Si-PDIA6. Compared to that in the common supernatant stimulation, PDIA6 expression in BMDCs was downregulated after knockdown (Fig. S5A, B). Meanwhile, the RNA expression and proportion of CD86, CD40, and MHCII in BMDCs stimulated by the pretreated supernatant (TM/hypoxia) were increased (Fig. S5C, D, F, G). Moreover, After PDIA6 was knocked down, BMDCs were co-cultured with CD8^+^ T cells. It was observed the proportion of INF-γ and the secretion of Granzyme B were increased in T cells (Fig. S5E, H). The above results suggest that ERS could promote the expression of PDIA6 in DC by increasing lactate level in the tumor microenvironment, thereby inhibiting the maturation and activation of DC and downregulating the anti-tumor activity of T cells.

### PDIA6 expression induced by ERS promotes the growth of gastric cancer and regulates the immune function of DC

**Fig. 7:**
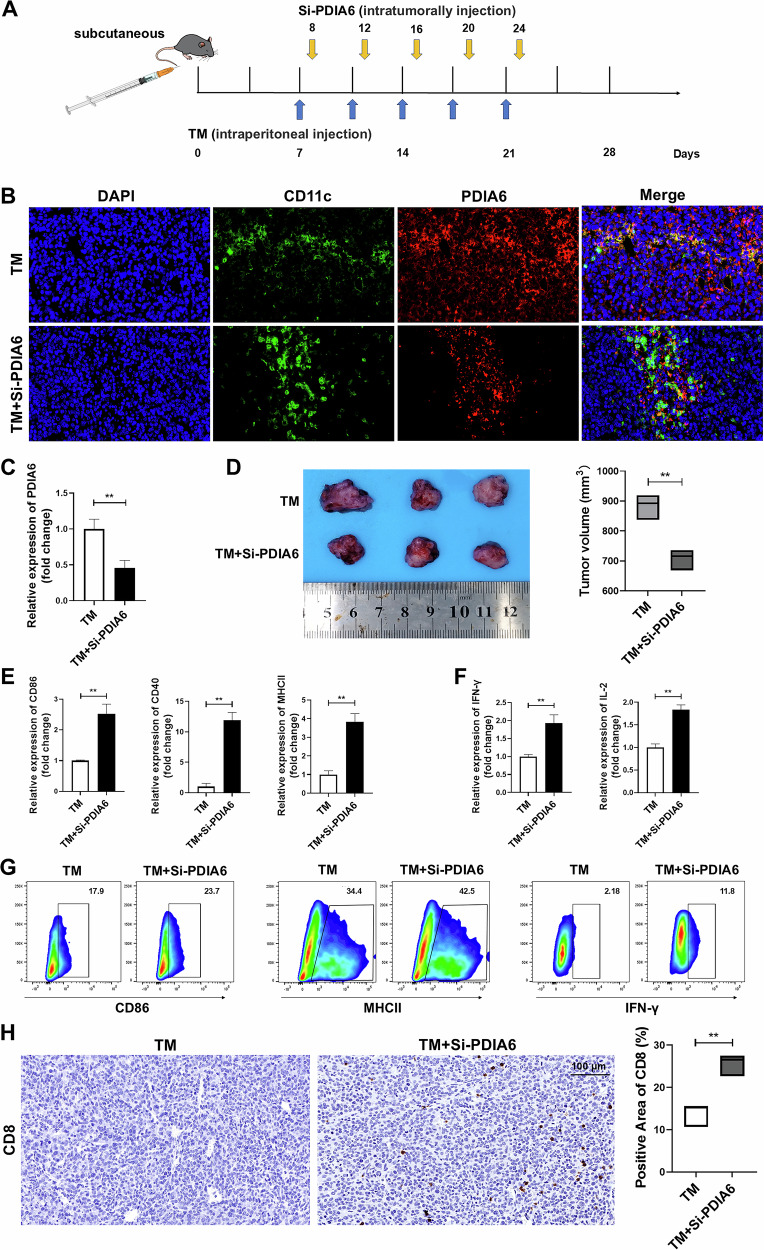
ERS promotes gastric cancer progression by upregulating PDIA6 level. **A** Subcutaneous tumor mice in ERS status was treated by Si-PDIA6: mice were subcutaneously injected with MFC on day 0, intraperitoneally injected with TM on day 7, as well as intratumorally injected with Si-PDIA6. After 28 days, the mice were euthanized. **B** Representative immunofluorescence staining was used to detect the expression of PDIA6 in DC. **C** RT-qPCR was used to detect the RNA level of PDIA6 in tissues. **D** The subcutaneous tumor was taken and photographed, and the tumor volume was calculated. **E**, **F** After using Si-PDIA6, RT-qPCR was used to detect the RNA expression level of CD86, CD40, MHCII, IFN-γ, and IL-2 in mice subcutaneous tumor tissue. **G** Flow cytometry was used to detect the proportion of CD86, MHCII, and IFN-γ. **H** Immunohistochemistry was used to detect the distribution of CD8^+^ T cells. *N* = 3, data expressed as Mean ± SD for different groups. *P < 0.05, **P < 0.01.

To test the effect of PDIA6 on gastric cancer, MFC were subcutaneously injected into mice. TM was injected intraperitoneally into the mice and Si-PDIA6 was injected intratumorally once every 4 days. After 28 days, the mice were sacrificed under anesthesia (Fig. 7A). We found that compared to that in the TM group, the protein and RNA expression of PDIA6 in the TM+Si-PDIA6 group was decreased (Fig. 7B, C), and the tumor volume in the TM+Si-PDIA6 group was also reduced (Fig. 7D).

To investigate the effect on immune cells of PDIA6 increase in gastric cancer, we detected the expression level of CD86, CD40, MHCII, IFN-γ and IL-2 in mice subcutaneous tumor tissue. The results showed that compared with that in the TM group, the expression level of these molecular markers was increased in the TM+Si-PDIA6 group (Fig. 7E, F). Flow cytometry results showed that the expression change of CD86, MHCII, and IFN-γ showed the same trend (Fig. 7G). We also used immunohistochemistry to detect the distribution of CD8^+^ T cells in the subcutaneous tumor tissue. The distribution of CD8^+^ T cells in the TM+Si-PDIA6 group was higher than that in the TM group (Fig. 7H). These results suggest that ERS could affect the maturation and activation of DC through the expression of PDIA6 and inhibit T cells from playing a role in killing, which promotes immune escape.

To evaluate the level of ERS response in gastric cancer, we collected tumor tissue samples from 16 gastric cancer patients. Initially, we performed immunohistochemistry to assess GRP78 expression in the tumor tissues and categorized the samples into two groups: low expression and high expression, based on the expression level (Fig. S6A). Next, we examined the distribution of CD8^+^ T cells and found that the distribution of CD8^+^ T cells was significantly higher in the GRP78 low expression group compared to the high expression group (Fig. S6B). This suggests that ERS may inhibit the proliferation of CD8^+^ T cells, contributing to an immunosuppressive microenvironment in gastric cancer.

To further investigate the relationship between ERS and H4K12 lactylation, we used immunofluorescence to measure H4K12 expression in gastric cancer tissues. We observed that the H4K12 level was significantly higher in the GRP78 high expression group than in the low expression group (Fig. S6C). Additionally, PDIA6 expression was also significantly elevated in the GRP78 high expression group (Fig. S6C). The expression trends of both H4K12 and PDIA6 were consistent, suggesting a potential regulatory connection between the two. More importantly, the expression level of GRP78 and PDIA6 were positively correlated in gastric cancer samples (Fig. S6D). Interestingly, the survival rate demonstrated a marked increase in PDIA6 low expression group compared with PDIA6 high expression group (Fig. S6E). These findings indicate that the ERS response in the gastric cancer microenvironment may be linked to H4K12 lactylation and could regulate the expression of PDIA6, thereby affecting the growth of gastric cancer.

## Discussion

As gastric cancer progresses, the tumors located in the cardia are more likely to cause dysphagia, whereas those located in the pylorus manifest as persistent vomiting [27]. In the late stage, upper abdominal pain intensifies, and symptoms such as bloody vomiting and black stools appear [4]. This indicates the urgent need to elucidate the underlying mechanisms and discover additional or alternative treatment methods for gastric cancer. Currently, radical surgery is the conventional treatment for gastric cancer [28]. Radiotherapy, chemotherapy, and targeted therapy are mainly used to treat advanced gastric cancer [29]. However, these treatment methods have limited effectiveness and are accompanied by a series of side effects that may be caused by the complex tumor microenvironment. Presently‌, numerous studies have indicated that ERS in the tumor microenvironment can facilitate immune escape in gastric cancer. However, the precise molecular mechanism by which ERS mediates immune escape within the gastric cancer microenvironment remains under investigation. In this study, we demonstrated that ERS-mediated lactate production inhibits the immune function of DC and contributes to immune escape in gastric cancer. The principal findings are as follows: ERS inhibits DC from expressing co-stimulatory molecules and CD8^+^ T cells from expressing cytokines by promoting PDIA6 expression through lactate, thereby accelerating the growth of gastric cancer. Our results highlight the importance of lactylation modification for the immunomodulatory properties of DC in ERS-mediated gastric cancer.

Numerous studies have shown that ERS is associated with gastric cancer development. Generally, the expression of ERS-related proteins is closely related to the development of malignant tumor and positively correlated with tumor size, depth of invasion, poor differentiation, tumor-lymph node metastasis stage, lymphatic and venous invasion, time to recurrence, and chemotherapy resistance [30]. Our results showed that tumor volume and GRP78 expression significantly increased after TM pretreatment.

DC, the most important antigen-presenting cell, can control immune response. DC can help reduce tumor generation and development [31, 32]. However, when the tumor microenvironment persists for a long time, the function of DC was gradually weakened [33, 34]. The important role of DC in tumor formation has been established for many years; however, the mechanism by which DC directly interact with the tumor microenvironment remains elusive. Reportedly, ERS can act on immune cells in the tumor microenvironment to exert immunosuppressive functions [35, 36]. For example, ERS plays a crucial role in promoting abnormal lipid accumulation in dysfunctional DC [37]. Our in vitro co-culture experiments also showed that ERS reduced the level of the co-stimulators CD86, CD40, and MHCII, as well as attenuated the killer factors of T cells, namely IFN-γ and Granzyme B. Thus, our data suggest that ERS downregulates the antitumor potential by impairing the immune function of DC.

The emergence of lactylation has increased our understanding of lactate function and its role in various pathophysiological conditions. For example, it can activate the transcription of multiple glycolysis-related genes in microglia in Alzheimer’s disease to promote glycolysis [38] and enhance demethylation, thereby accelerating the occurrence of ocular melanoma tumors [39]. In this study, we demonstrated that ERS promoted the growth of gastric cancer by promoting lactate secretion by tumor cells. Based on this, we further explored the effect of ERS on H4K12 lactylation levels in DC by upregulating the lactate content in the tumor microenvironment. The endoplasmic reticulum chaperone, PDIA6, is a member of the protein disulfide isomerase family A. PDIA6 is found mainly in the endoplasmic reticulum, where it catalyzes the formation of disulfide bonds and promotes protein folding. PDIA6 plays a crucial role in cancer progression [40, 41]. For example, it facilitates immune escape in pancreatic cancer cells because it can promote tumor progression by facilitating aerobic glycolysis, thereby increasing lactate production [42]. It also promotes aerobic glycolysis in oral squamous cell carcinoma and increases lactate production, thereby promoting tumor progression [43]. Further studies on H4K12 and PDIA6 showed striking finding that ERS promotes the expression of PDIA6 through H4K12 lactylation to inhibit DC maturation and T cell activation, thus mediating immune escape in gastric cancer.

**Fig. 8:**
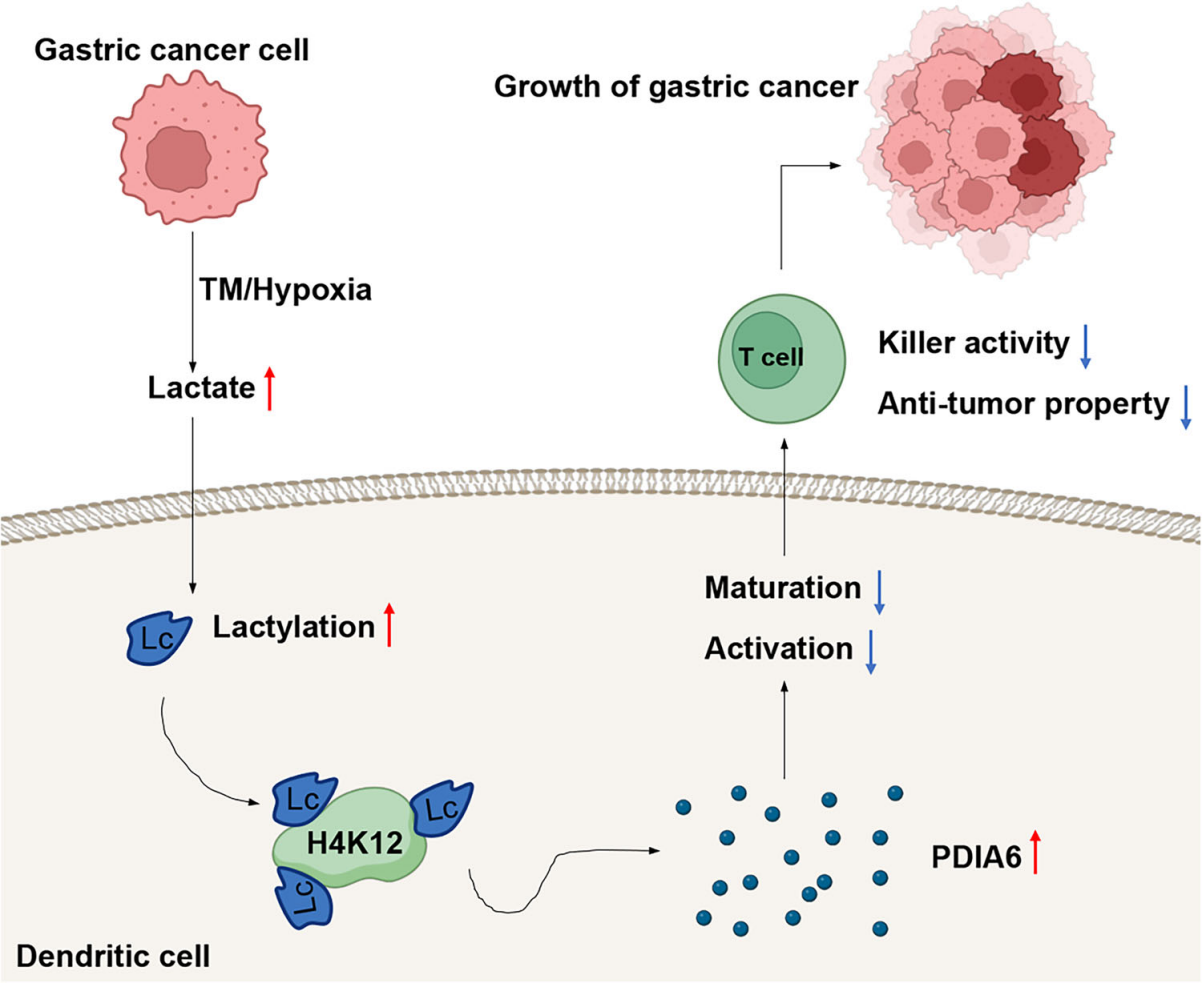
ERS-induced lactylation in DC drives immune escape via PDIA6 in gastric cancer. Schematic: ERS mediated-elevation of lactate in tumor cells leads to lactylation in DC and promotes the expression of PDIA6, thereby causing immune escape in gastric cancer.

With changes in the living environment and increased social pressure, the incidence of gastric cancer has increased annually. Nevertheless, immune regulatory points targeting the tumor microenvironment are insufficient, and many immunotherapies have not yet entered the clinical stage. Therefore, exploring potential molecular mechanisms for controlling immune targets has become one of the main challenges in gastric cancer immunotherapy. Figure 8 illustrates the molecular mechanism through which ERS mediates immune escape in gastric cancer by suppressing DC function. ERS contributes to the H4K12 lactylation of DC by promoting the secretion of lactate from gastric cancer cells, thereby upregulating the expression of PDIA6, inhibiting the expression of DC-related markers, and weakening the activation of T cells.

Finally, there are still some limitations in our study. While our study establishes a crucial link between ERS- H4K12-PDIA6 axis, and DC dysfunction, our findings primarily rely on models of acute ERS. Chronic ERS could elicit cell-autonomous effects in tumors. Moreover, whether the mutation of the H4K12 lactylation site in vivo could lead to the weakening of DC function and the growth of tumors remains unknown and is worthy of investigation. Therefore, we will use mouse models (such as XBP1 gene knockout mice and H4K12R gene knock-in) for further in vivo research to better elucidate in tumor the role of ERS-H4K12-PDIA6 axis on DC.

## Conclusion

Our study demonstrates that ERS can drive lactate accumulation in gastric cancer cells, thereby increasing the abundance of histone lactylation modification in DC. We also found that the H4K12 site undergoes lactylation modification and can promote the expression of the endoplasmic reticulum-related protein PDIA6, thereby inhibiting the immune response of DC and downregulating T cells activation. Therefore, this histone lactylation site could serve as a new biomarker for immunotherapies that alter DC function in gastric cancer.

## Supplementary information

Figure S1

Figure S2

Figure S3

Figure S4

Figure S5

Figure S6

Supplement figure legends

Full and uncropped western blots

supplementary materials

## Data Availability

All data generated and/or analyzed during this study are included in this published article. The mass spectrometry proteomics data have been deposited to the ProteomeXchange Consortium (https://www.iprox.cn/) via the iProX proteomics database with the project PXD072553. Transcriptome sequencing data have been deposited to the NCBI (https://www.ncbi.nlm.nih.gov/) with PRJNA1395301.
