# Supplementary material for: Endoplasmic reticulum stress-induced histone lactylation mediating immune escape of gastric cancer via PDIA6 overexpression in dendritic cells
Source: Cell Death Dis. 2026 Mar 27;17(1):686. doi: 10.1038/s41419-026-08638-9 (PMC13444140; doi:10.1038/s41419-026-08638-9)

fig S1

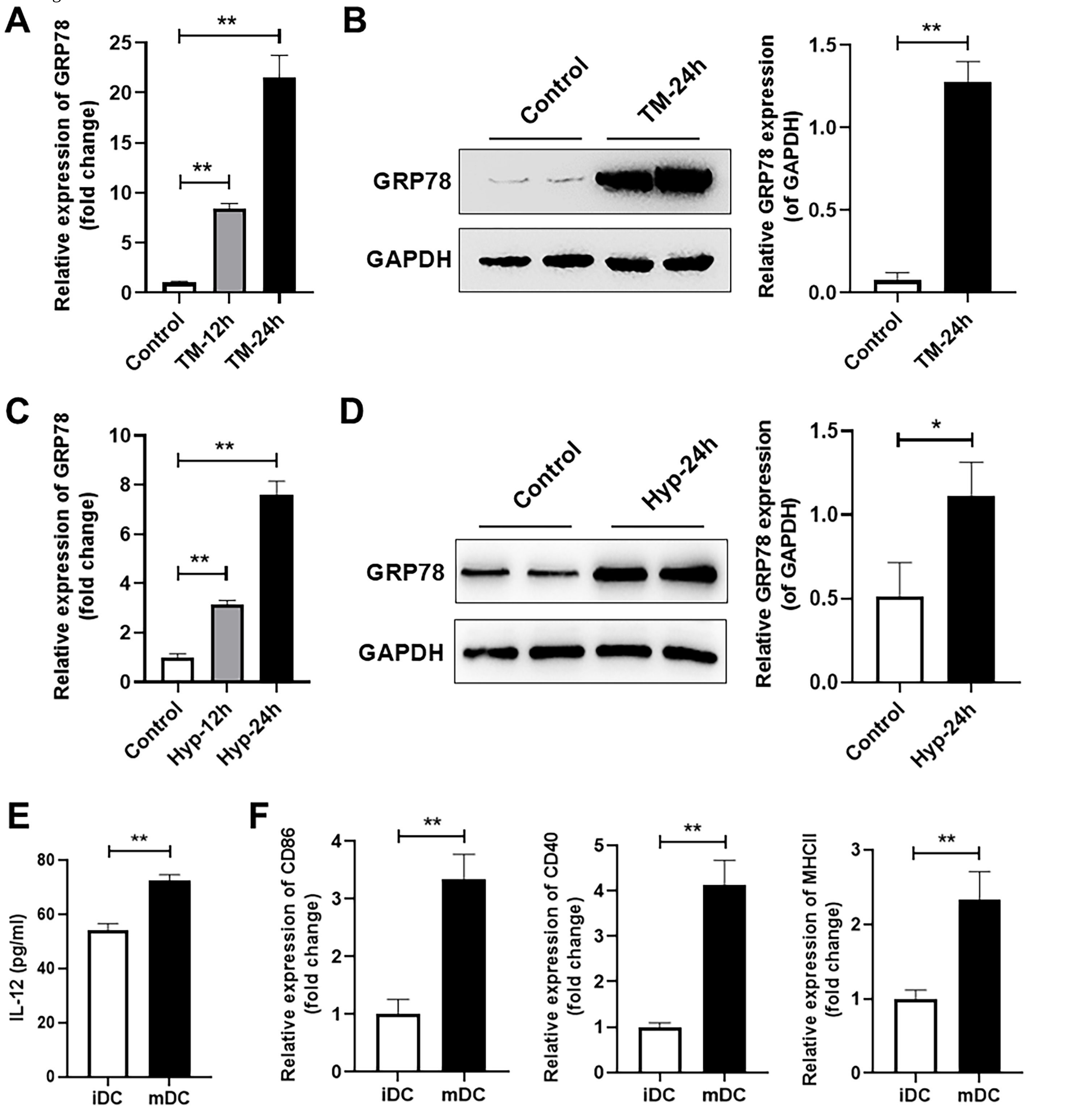
A
Relative expression of GRP78 (fold change)
25
20
15
10
5
0
**
**
Control
TM-12h
TM-24h
B
Control
TM-24h
GRP78
GAPDH
Relative GRP78 expression (of GAPDH)
1.5
1.0
0.5
0.0
**
Control
TM-24h
C
Relative expression of GRP78 (fold change)
10
8
6
4
2
0
**
**
Control
Hyp-12h
Hyp-24h
D
Control
Hyp-24h
GRP78
GAPDH
Relative GRP78 expression (of GAPDH)
1.5
1.0
0.5
0.0
*
Control
Hyp-24h
E
IL-12 (pg/ml)
80
60
40
20
0
**
iDC
mDC
F
Relative expression of CD86 (fold change)
4
3
2
1
0
**
iDC
mDC
Relative expression of CD40 (fold change)
5
4
3
2
1
0
**
iDC
mDC
Relative expression of MHCII (fold change)
3
2
1
0
**
iDC
mDC

fig S2

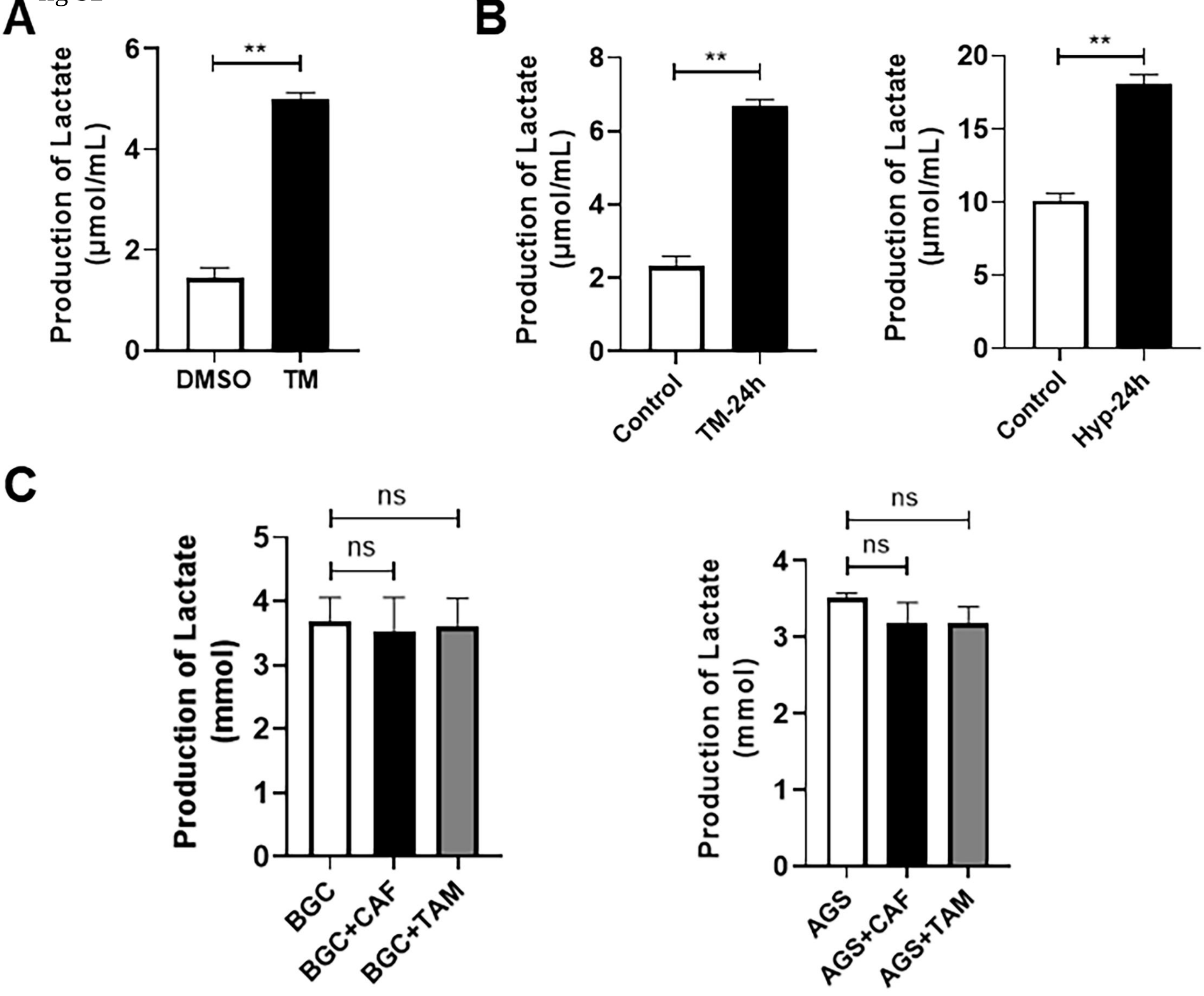

A
Production of Lactate (μmol/mL)
0
2
4
6
**
DMSO
TM
B
Production of Lactate (μmol/mL)
0
2
4
6
8
**
Control
TM-24h
Production of Lactate (μmol/mL)
0
5
10
15
20
**
Control
Hyp-24h
C
Production of Lactate (mmol)
0
1
2
3
4
5
ns
ns
BGC
BGC+CAF
BGC+TAM
Production of Lactate (mmol)
0
1
2
3
4
ns
ns
AGS
AGS+CAF
AGS+TAM

fig S3

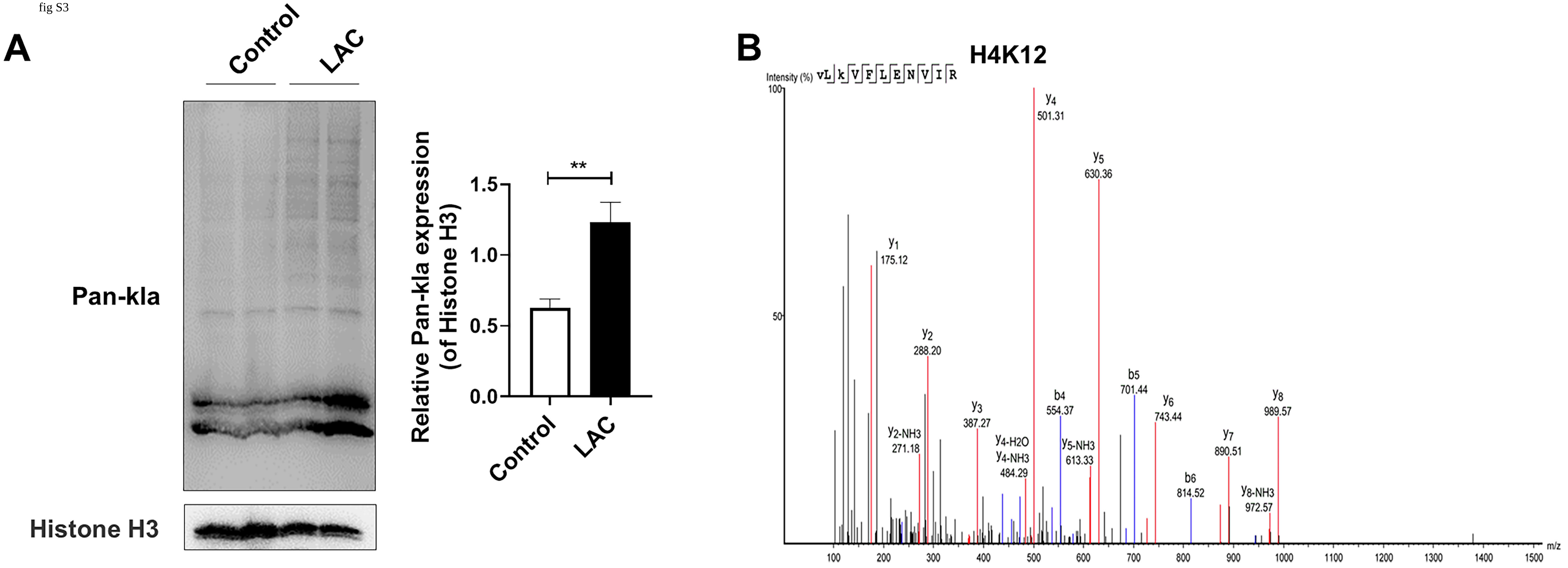
A
Control
LAC
Pan-kla
Histone H3
Relative Pan-kla expression (of Histone H3)
**
1.5
1.0
0.5
0.0
Control
LAC
B
H4K12
Intensity (%) vLkVFLENVIR
y4 501.31
y5 630.36
y1 175.12
y2 288.20
y3 387.27
y2-NH3 271.18
y4-H2O y4-NH3 484.29
b4 554.37
y5-NH3 613.33
b5 701.44
y6 743.44
b6 814.52
y7 890.51
y8-NH3 972.57
y8 989.57
m/z

fig S4

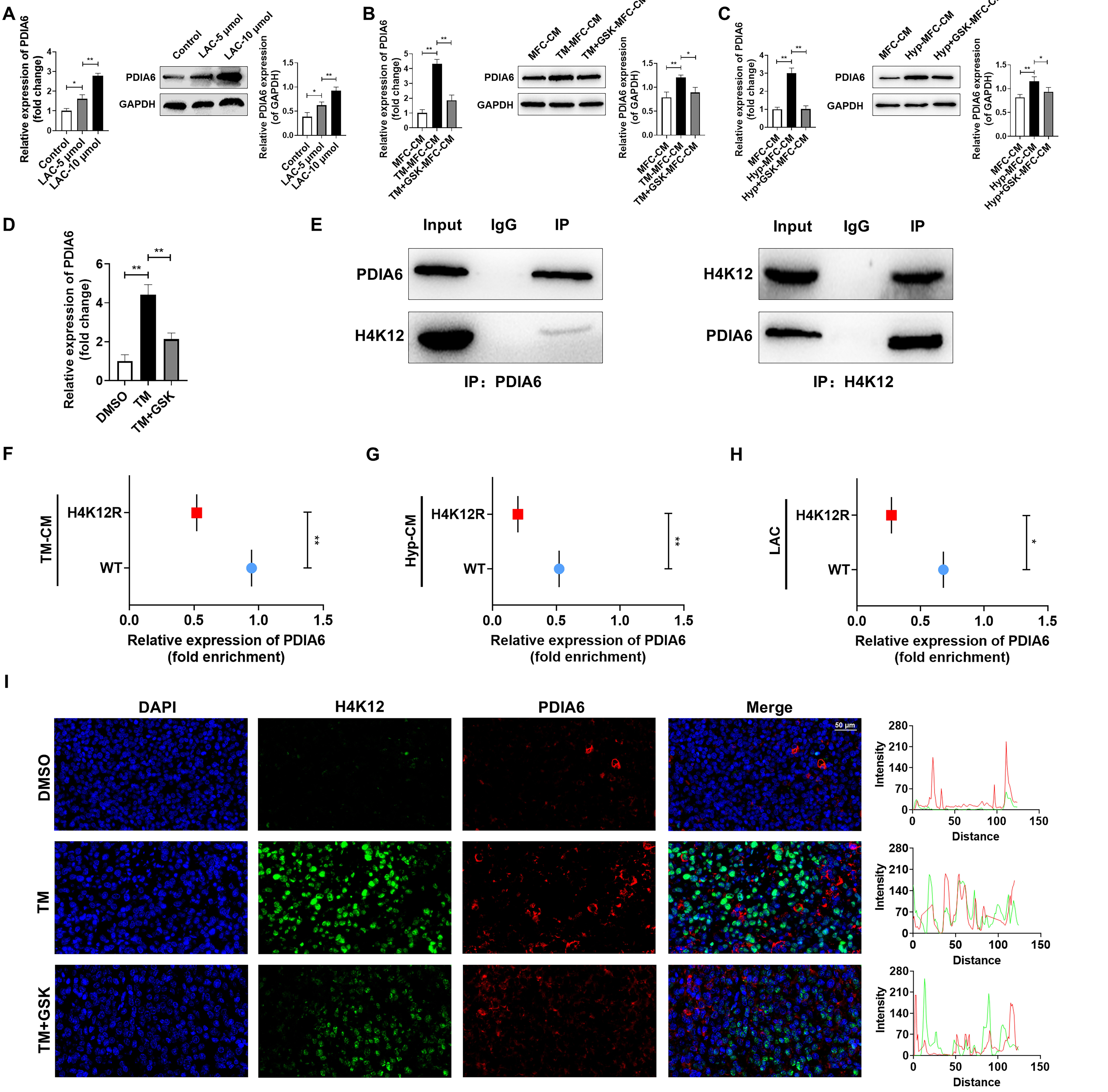

A
Relative expression of PDIA6 (fold change)
Control
LAC-5 μmol
LAC-10 μmol
PDIA6
GAPDH
Relative PDIA6 expression (of GAPDH)
B
MFC-CM
TM-MFC-CM
TM+GSK-MFC-CM
C
Hyp-MFC-CM
Hyp+GSK-MFC-CM
D
DMSO
TM
TM+GSK
E
Input
IgG
IP
H4K12
IP: PDIA6
IP: H4K12
F
TM-CM
H4K12R
WT
Relative expression of PDIA6 (fold enrichment)
G
Hyp-CM
H
LAC
I
DAPI
Merge
50 μm
Intensity
Distance

fig S5

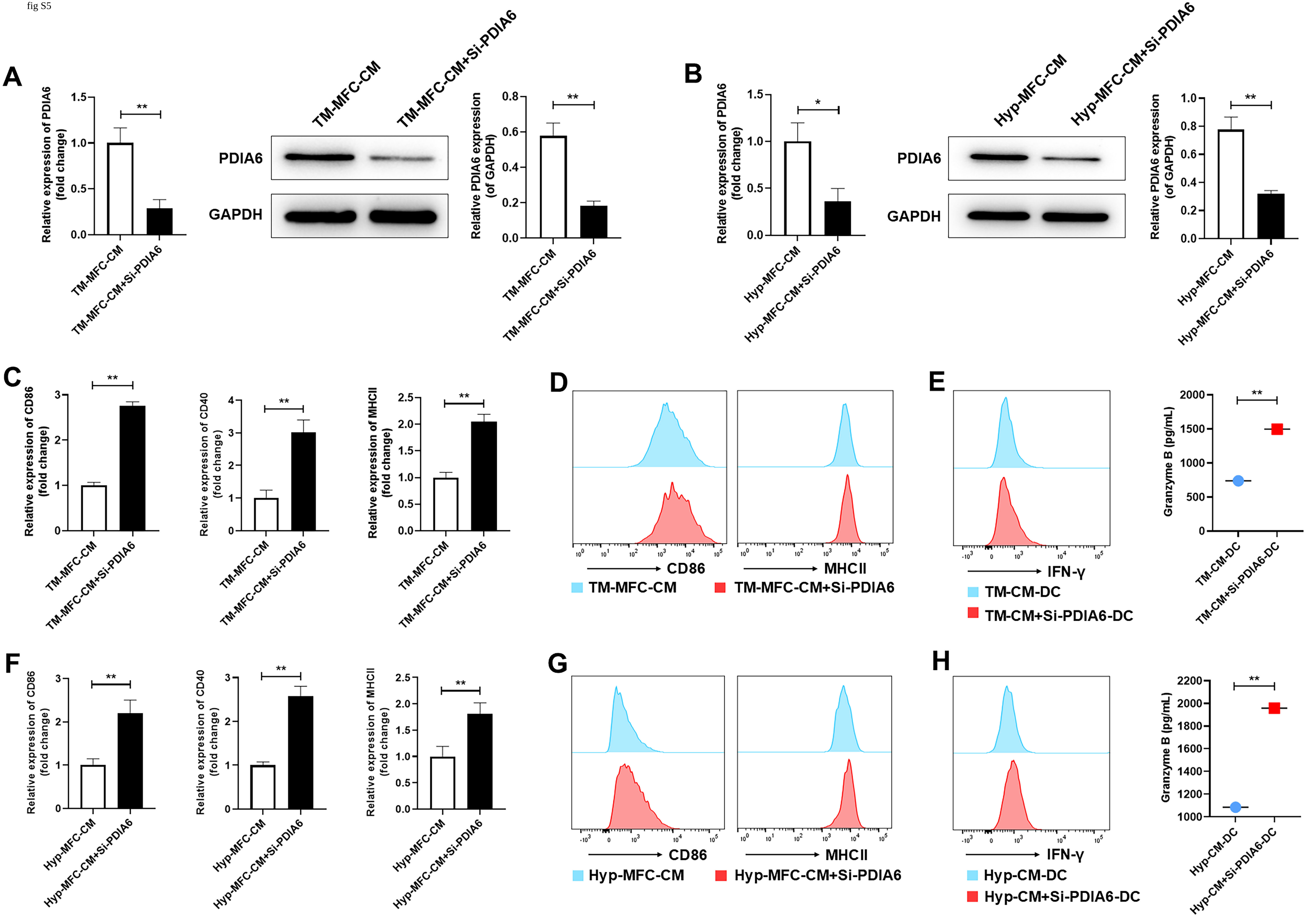

A
B
C
D
E
F
G
H
PDIA6
GAPDH
TM-MFC-CM
TM-MFC-CM+Si-PDIA6
Hyp-MFC-CM
Hyp-MFC-CM+Si-PDIA6
Relative expression of PDIA6 (fold change)
Relative PDIA6 expression (of GAPDH)
Relative expression of CD86 (fold change)
Relative expression of CD40 (fold change)
Relative expression of MHCII (fold change)
CD86
MHCII
IFN-γ
TM-CM-DC
TM-CM+Si-PDIA6-DC
Hyp-CM-DC
Hyp-CM+Si-PDIA6-DC
Granzyme B (pg/mL)

fig S6

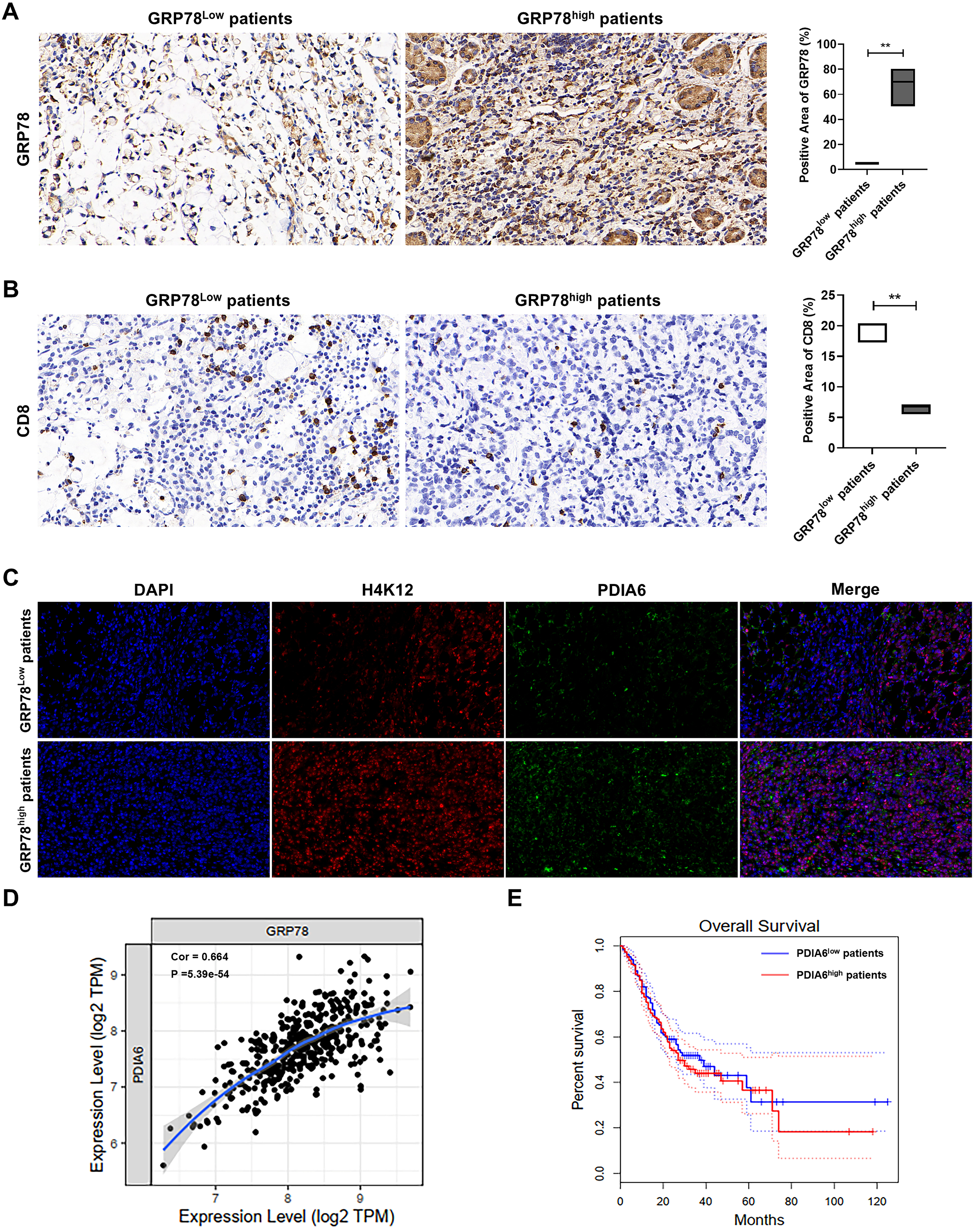
A
GRP78Low patients
GRP78high patients
GRP78
Positive Area of GRP78 (%)
**
GRP78low patients
GRP78high patients
B
GRP78Low patients
GRP78high patients
CD8
Positive Area of CD8 (%)
**
GRP78low patients
GRP78high patients
C
DAPI
H4K12
PDIA6
Merge
GRP78Low patients
GRP78high patients
D
GRP78
PDIA6
Cor = 0.664
P =5.39e-54
Expression Level (log2 TPM)
Expression Level (log2 TPM)
E
Overall Survival
PDIA6low patients
PDIA6high patients
Percent survival
Months

Fig. S1 Construction of ERS model of gastric cancer cells and obtainment of BMDCs.

(A, B) After TM stimulation of MFC, RNA and protein expression levels of GRP78 were detected. (C, D) After hypoxia stimulation, RNA and protein expression levels of GRP78 were detected. (E) ELISA was used to detect IL-12 secretion by iDC and mDC. (F) RT-qPCR was used to detect the RNA level of CD86, CD40, and MHCII. N = 3, data expressed as Mean ± SD for different groups. * P <0.05, ** P <0.01

Fig. S2 ERS promotes elevation of lactate level in gastric cancer cells.

(A) After using a lactate detection kit to treat subcutaneous tumor tissue in mice, the supernatant was collected to detect the lactate content in tumor cells. (B) After TM/hypoxia stimulation of MFC, the supernatant was collected to detect lactate secretion level. (C) BGC-823 and AGS were co-cultured with TAMs and CAFs respectively, then the supernatant was collected to detect lactate secretion level. N = 3, data expressed as Mean ± SD for different groups. * P <0.05, ** P <0.01

Fig. S3 The detection of histone lactylation at H4K12 sites in DC

(A) After BMDCs were treated with lactate, nuclear protein was extracted and the expression level of Pan-kla was detected by western blotting. (B) BMDCs histone protein was extracted. The modification was detected by mass spectrometry, and the secondary mass spectrometry peak showed lactylation of lysine at position 12. N = 3, data expressed as Mean ± SD for different groups. * P <0.05, ** P <0.01

Fig. S4 ERS enhanced the expression of PDIA6 via H4K12 lactylation in DC.

(A) BMDCs were stimulated by gradient lactate, then RNA and protein levels of PDIA6 were detected. (B, C) Pretreated-supernatant and GSK were used to stimulate BMDCs, then RNA and protein levels of

PDIA6 were detected. (D) GSK was treated with subcutaneous tumor mice in ERS status, then RNA level of PDIA6 was measured. (E) CO-IP experiment verified the relationship between H4K12 and PDIA6. (F, G, H) Lentivirus was used to infect BMDCs for H4K12 point mutation, then pretreated-supernatant/lactate were used to stimulate BMDCs, and ChIP-qPCR analysis of the indicated promoters was performed by using of H4K12 antibody. (I) GSK was treated with subcutaneous tumor mice in ERS status, and representative immunofluorescence staining was performed for H4K12 and PDIA6. N = 3, data expressed as Mean ± SD for different groups. * P <0.05, ** P <0.01

Fig. S5 ERS inhibits the immune function of DC by upregulating lactate level.

(A, B) Supernatant was added to BMDCs after Si-PDIA6 treatment, and the expression of PDIA6 was detected by RT-qPCR and western blotting. (C, F) RT-qPCR was used to detect the RNA expression level of CD86, CD40, and MHCII in BMDCs. (D, G) The proportion of CD86 and MHCII was detected using flow cytometry. (E, H) BMDCs were co-cultured with $CD8^+$ T cell, then the proportion of INF- γ and the secretion level of Granzyme B were measured. N = 3, data expressed as Mean ± SD for different groups. * P <0.05, ** P <0.01

Fig. S6 The role of ERS in gastric cancer microenvironment on the lactylation of H4K12 and PDIA6

(A) Immunohistochemical was used to detect the expression of GRP78 in gastric cancer tissue. (B) The distribution of $CD8^+$ T cell was detected according to GRP78 grouping. (C) Immunofluorescence double staining was used to detect the expression of H4K12 and PDIA6 in gastric cancer tissue. (D) Expression of GRP78 and PDIA6 in gastric cancer tissue was analyzed. (E) Overall survival curves for gastric cancer patients with different expression levels of PDIA6. N = 16, data expressed as Mean ± SD for different groups. * P <0.05， ** P <0.01

Supplement 2

Fig. 2A

GRP78

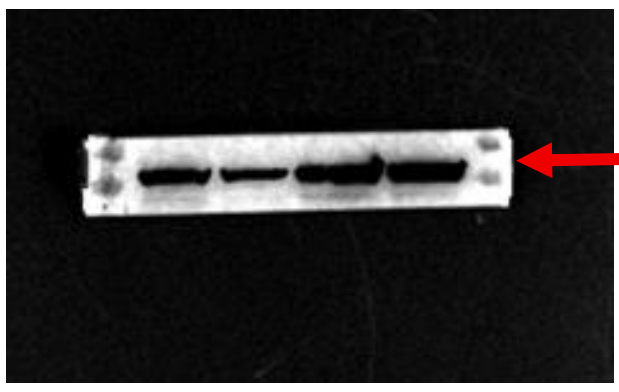

78KD

GAPDH

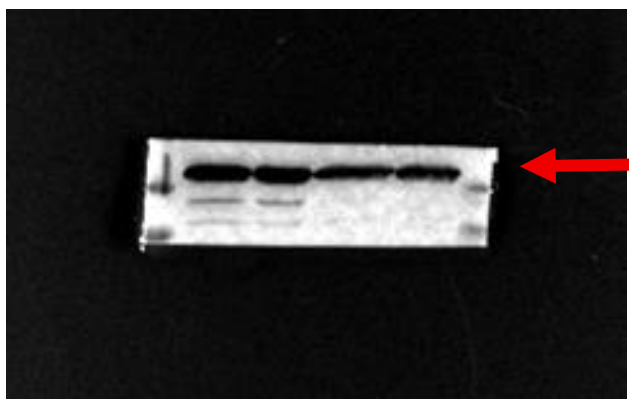

37KD

Fig. 2B

GRP78

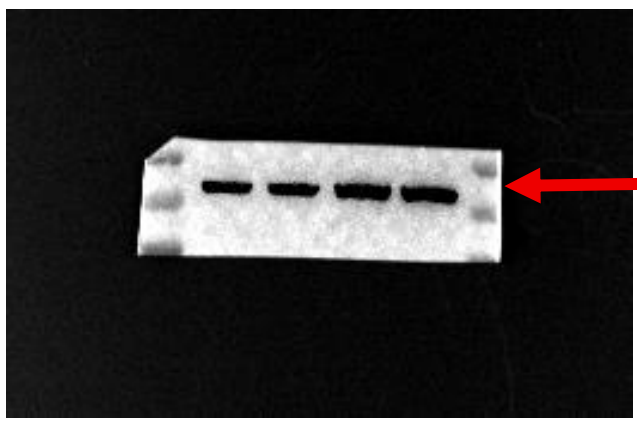

78KD

GAPDH

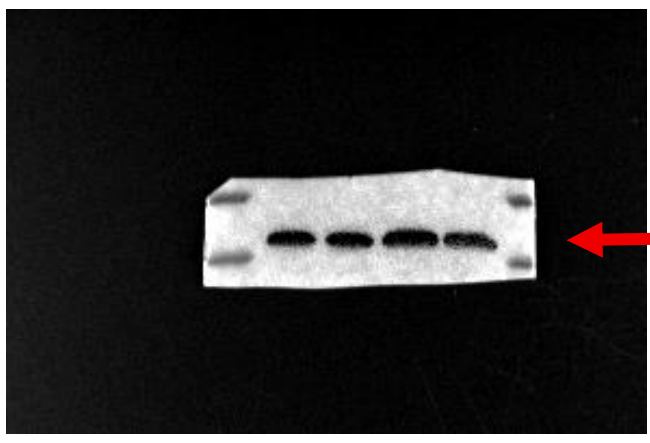

37KD

Fig. 5A

Pan-kla

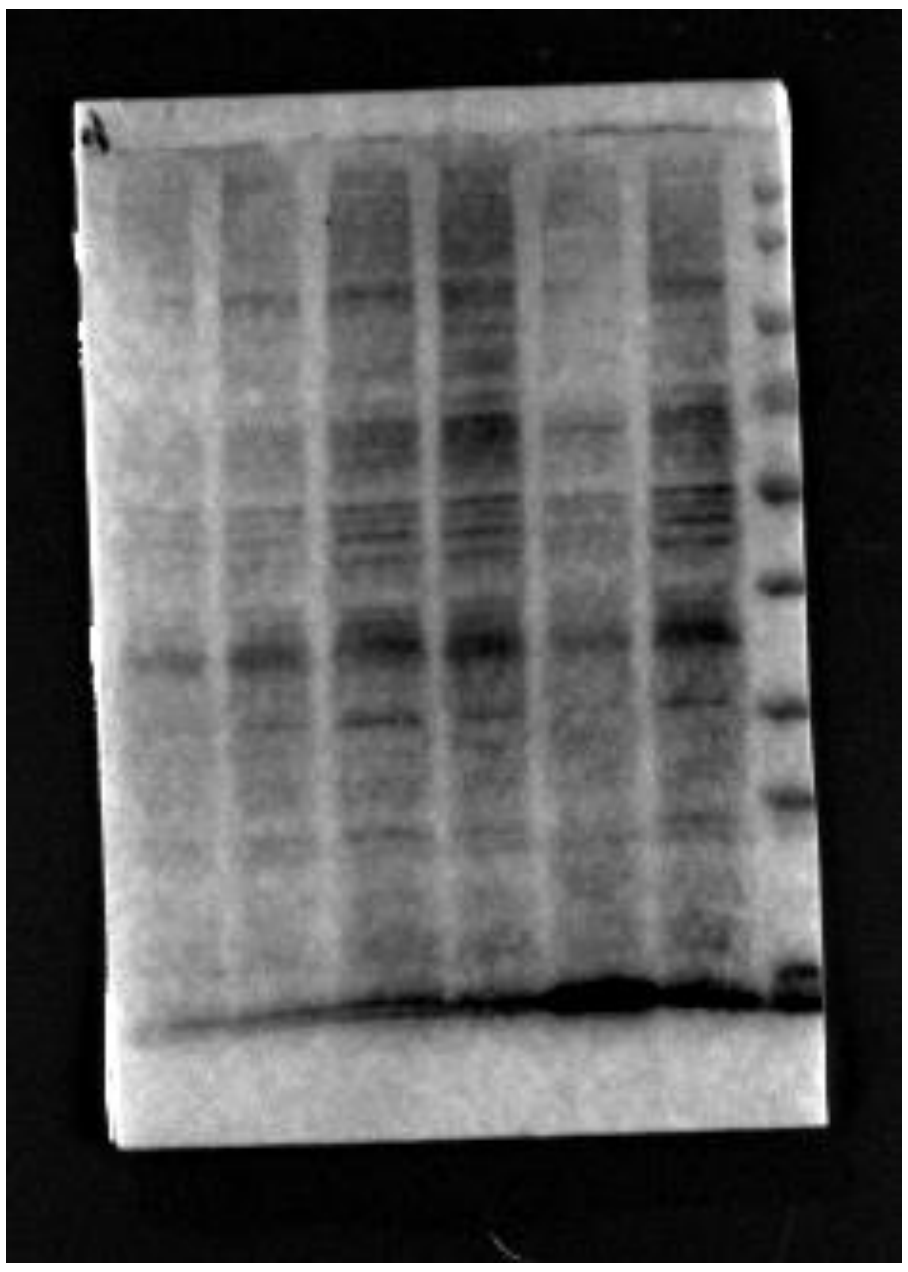

MFC-CM

TM-MFC-CM

GAPDH

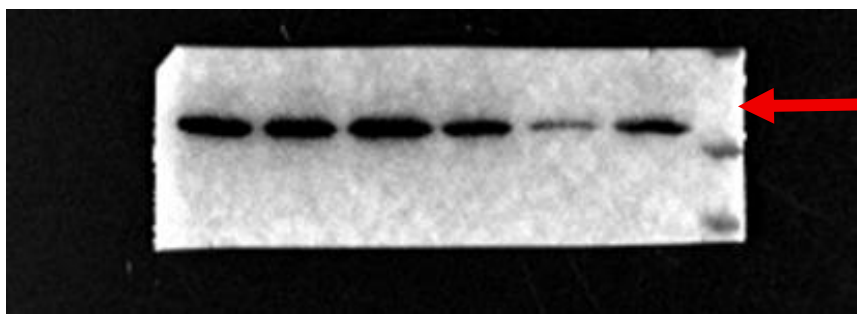

37KD

Fig. 5B

Pan-kla

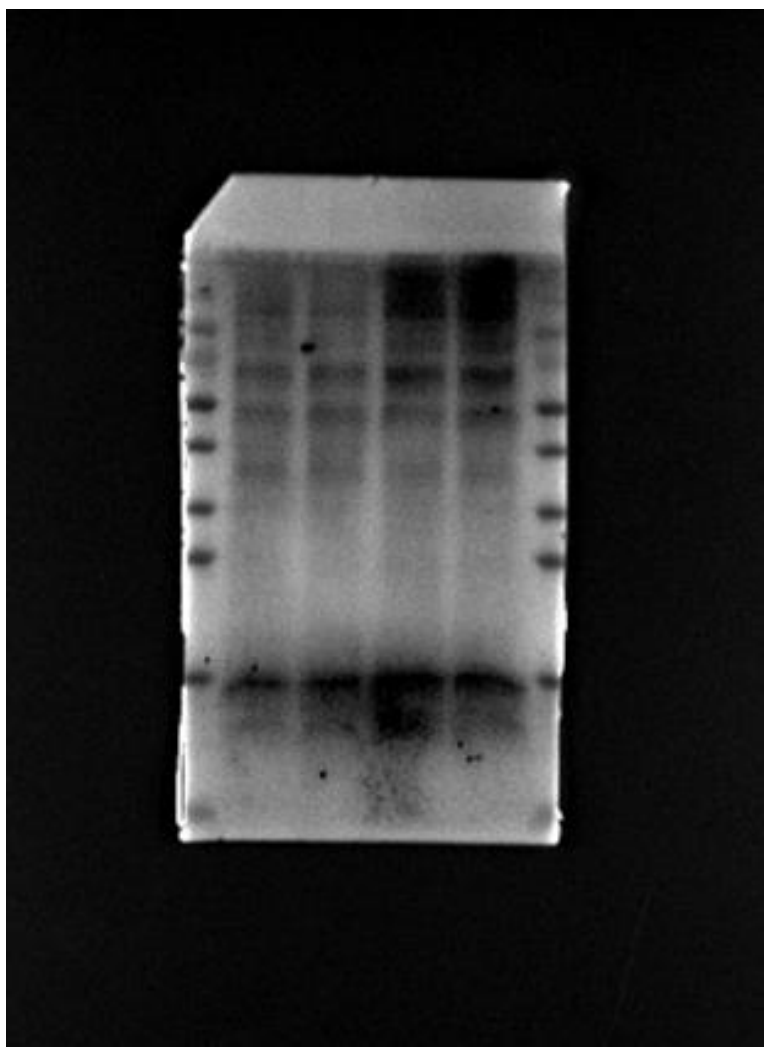

GAPDH

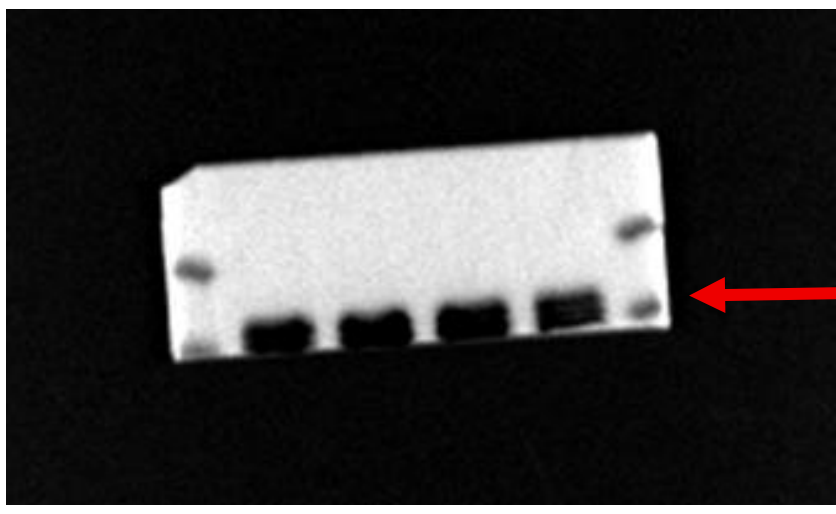

37KD

Fig. 5C

Pan-kla

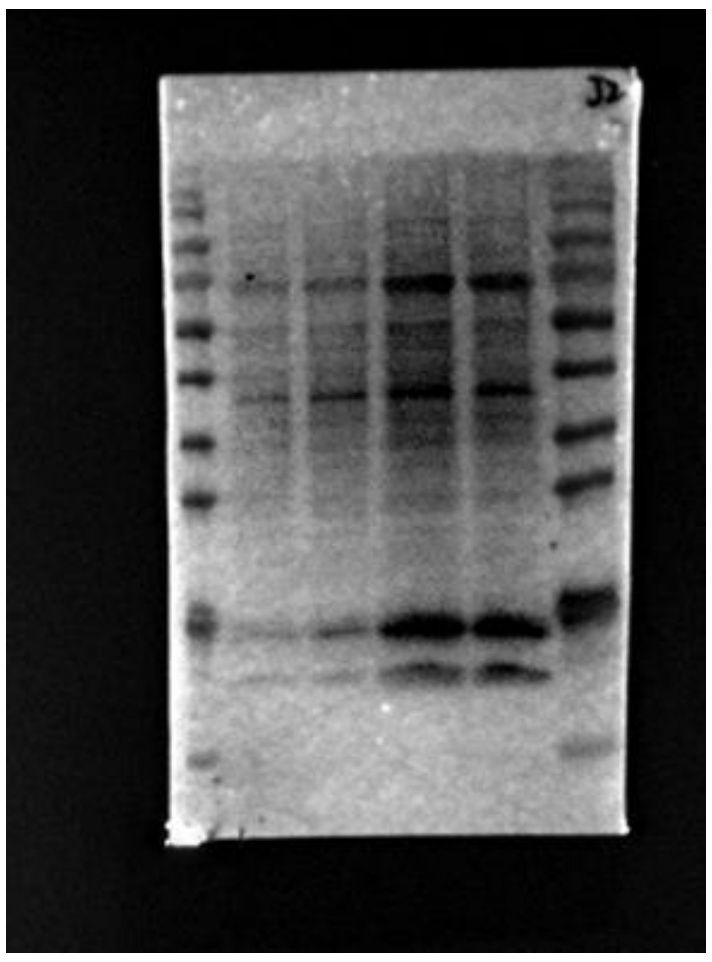

GAPDH

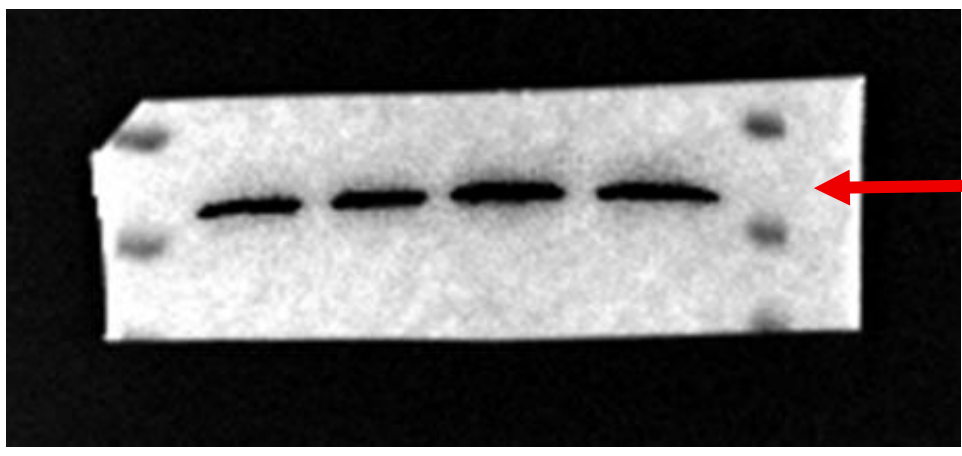

37KD

Fig. 5E

H4K12

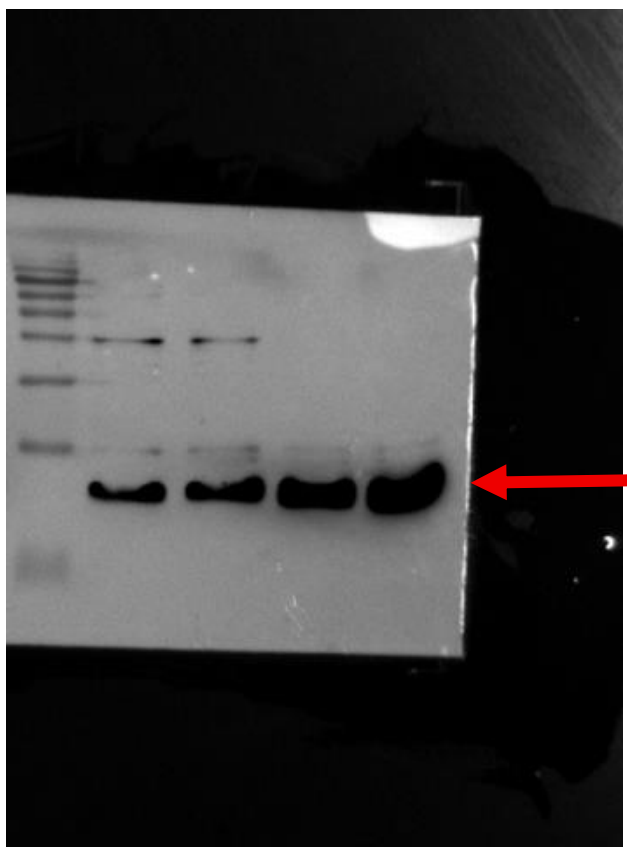

11KD

Histone H3

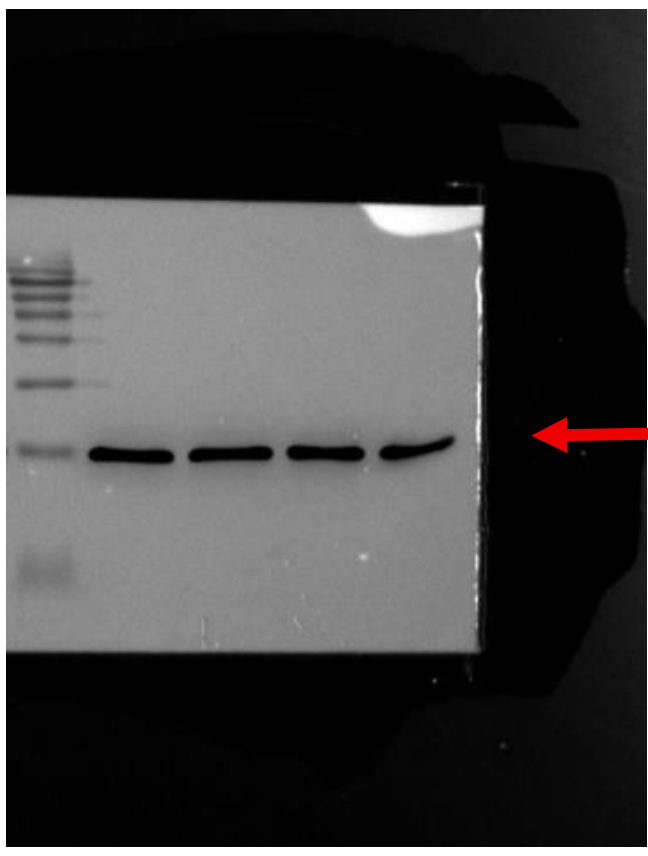

15KD

Fig. 5F

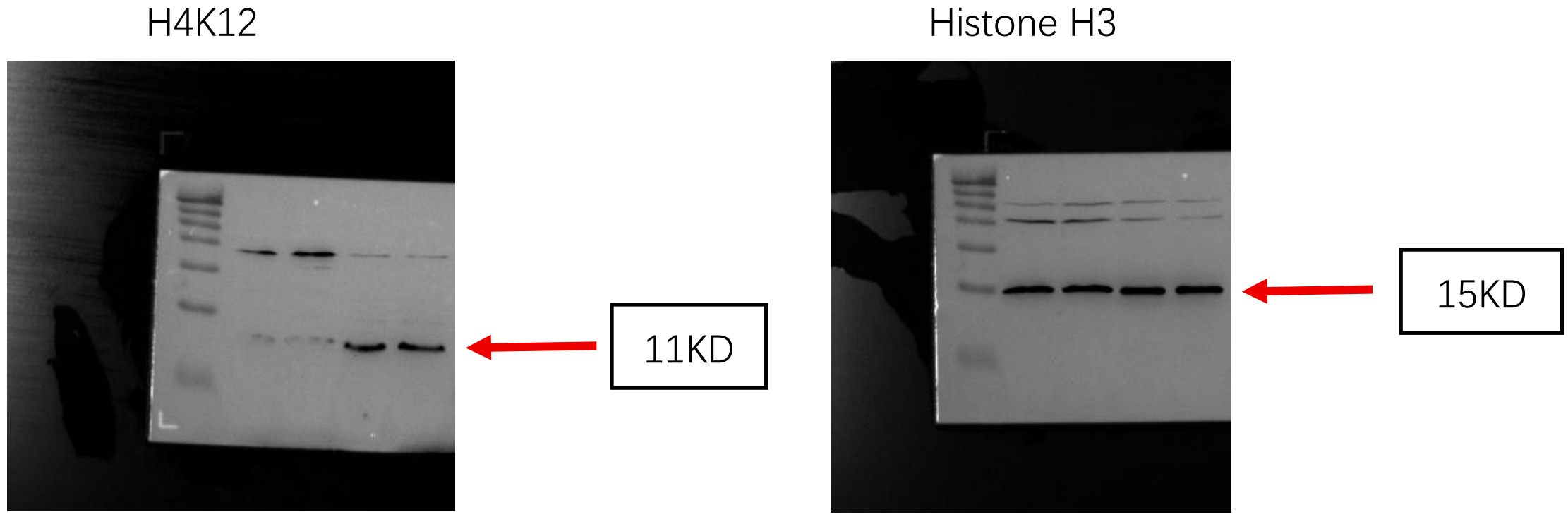

Fig. 5G

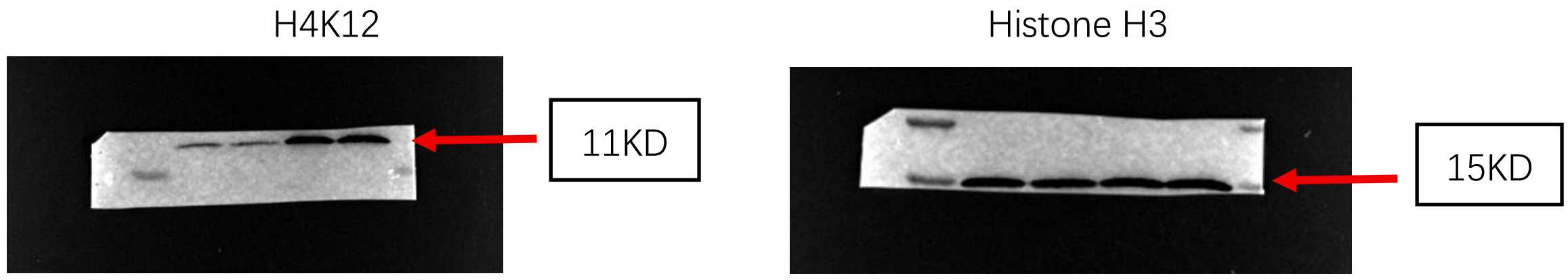

Fig. 6C

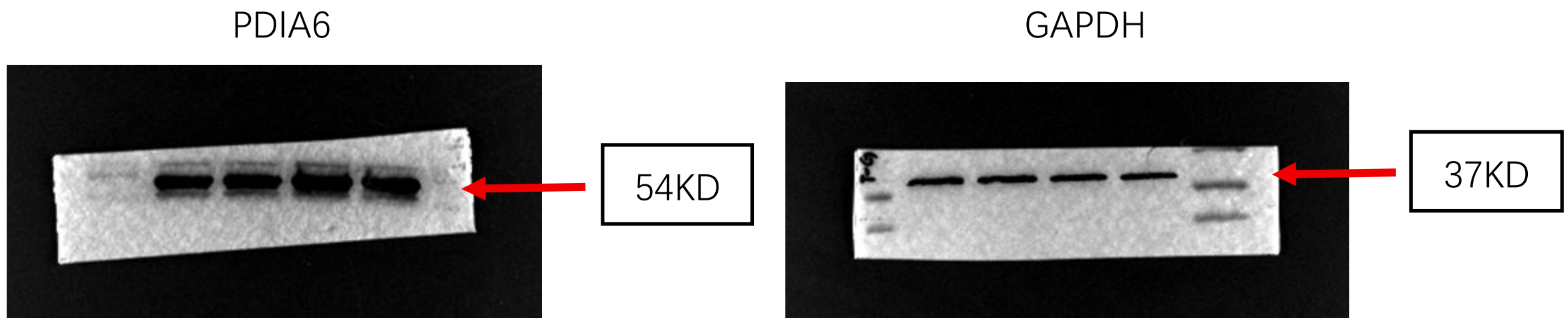

Fig. 6D

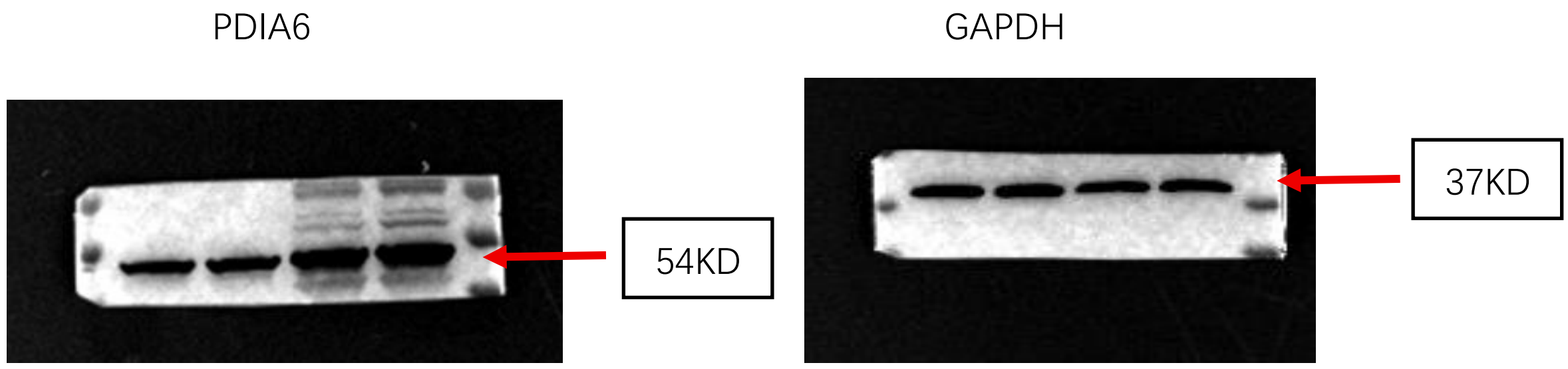

Fig. 6I

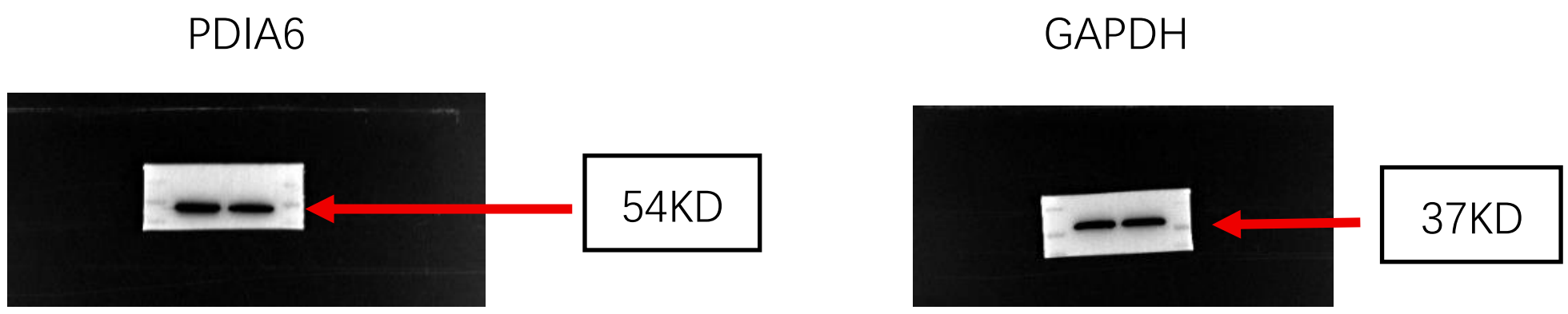

Fig. 6J

PDIA6

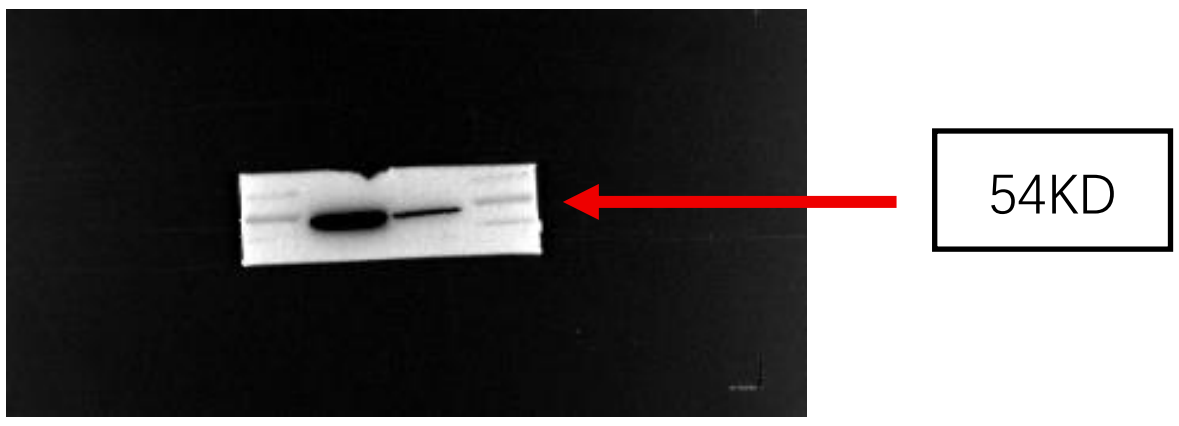

GAPDH

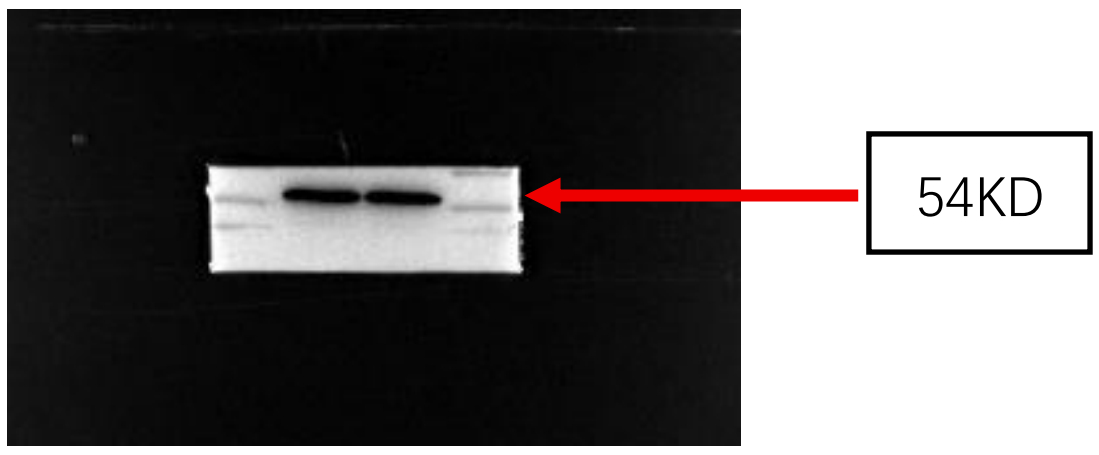

Fig. 6K

PDIA6

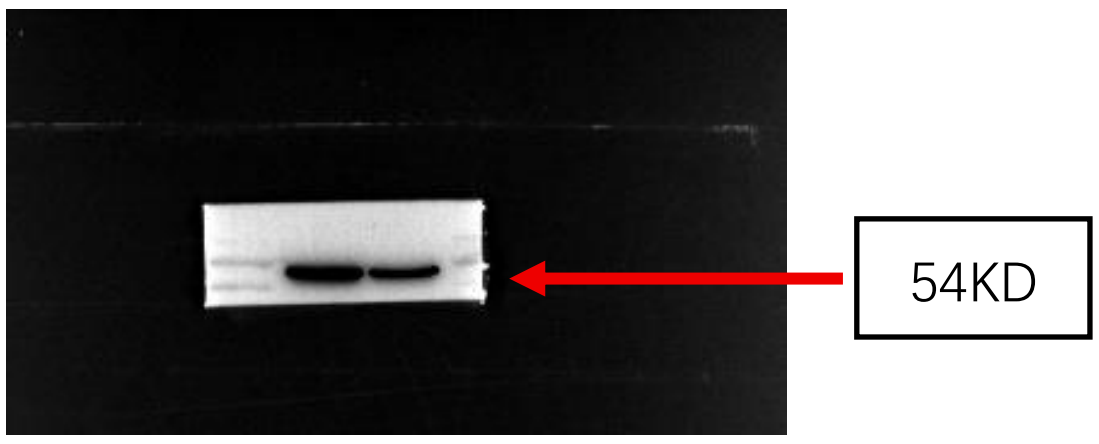

GAPDH

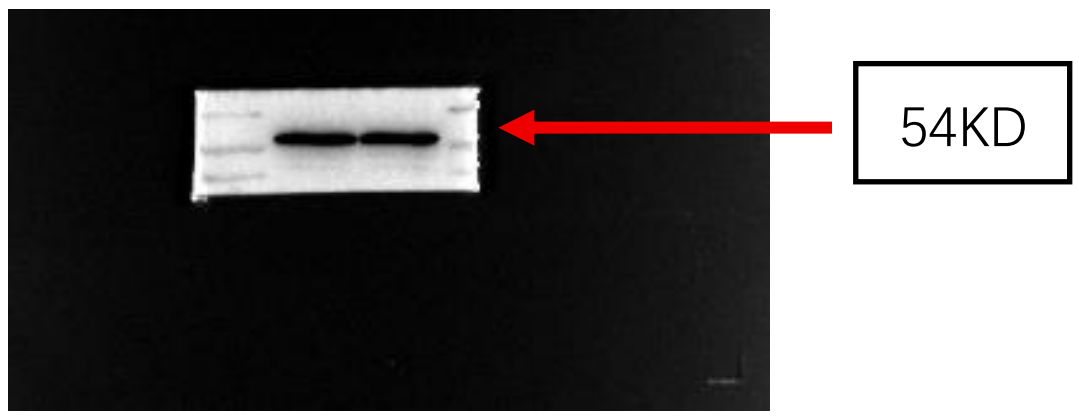

Fig. S1B

GRP78

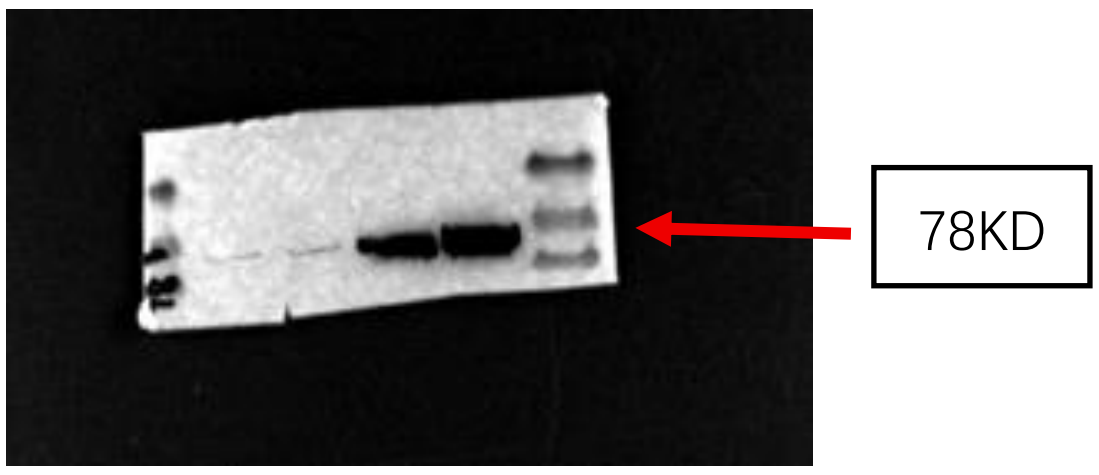

GAPDH

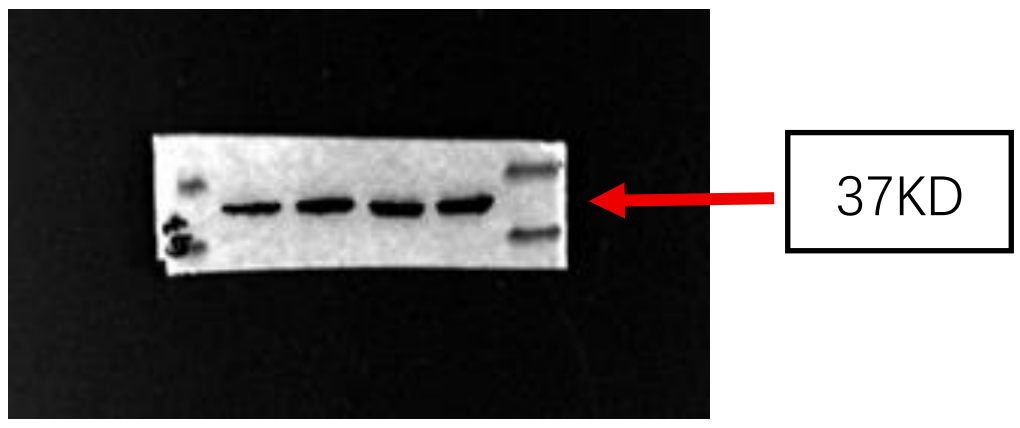

Fig. S1D

GRP78

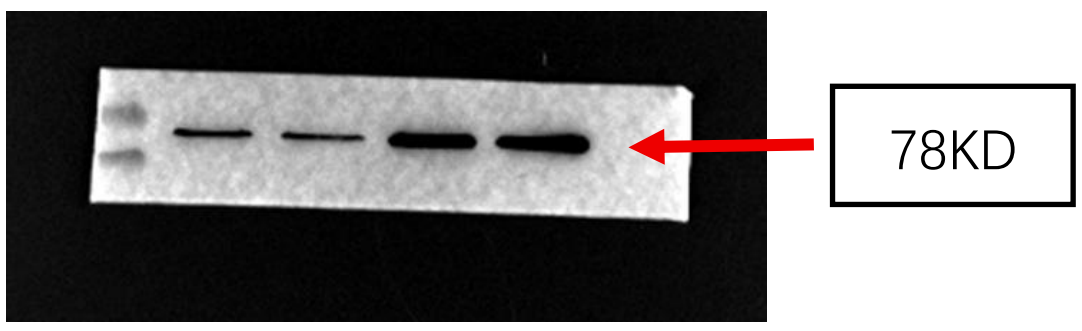

GAPDH

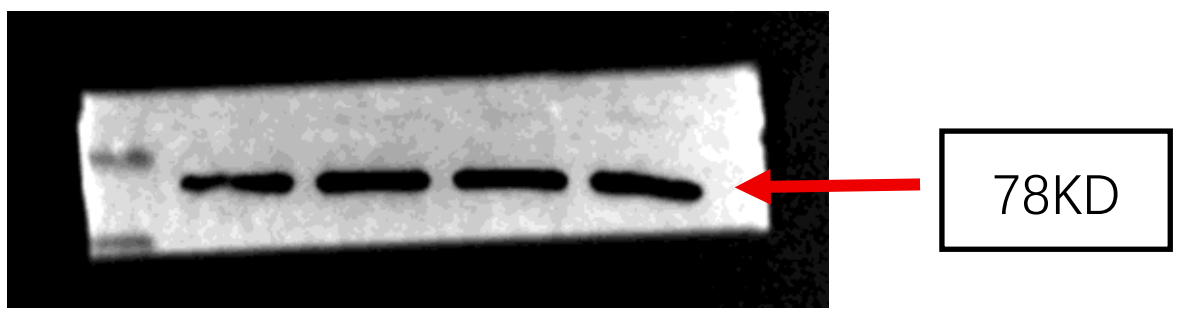

Fig. S3A

Pan-kla

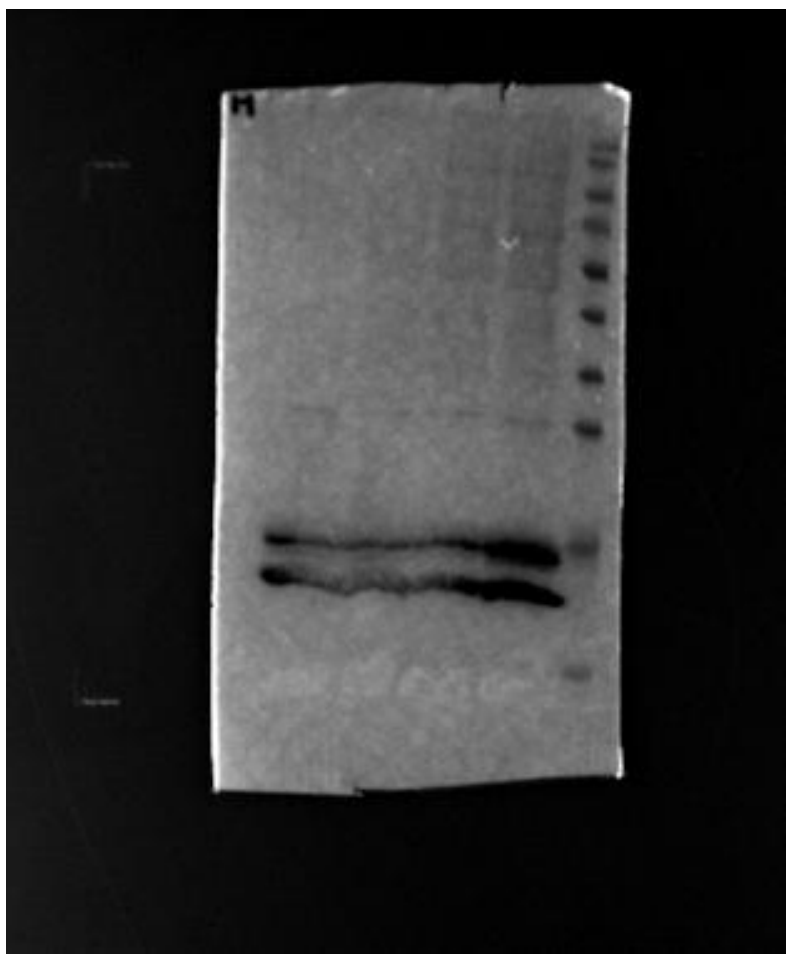

Histone H3

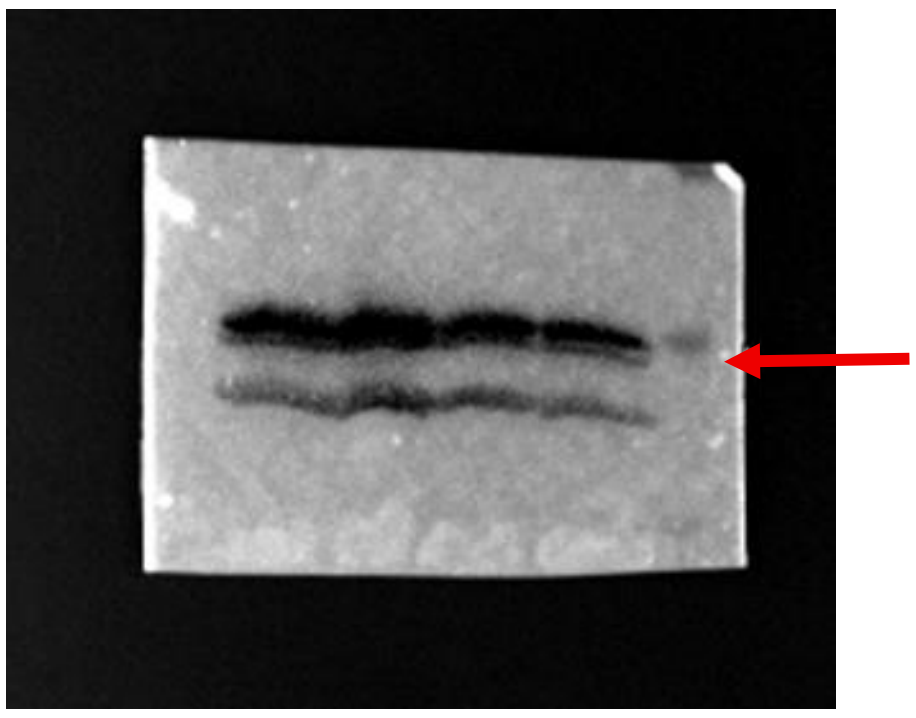

15KD

Fig. S4A

IP: PDIA6

Input IgG IP

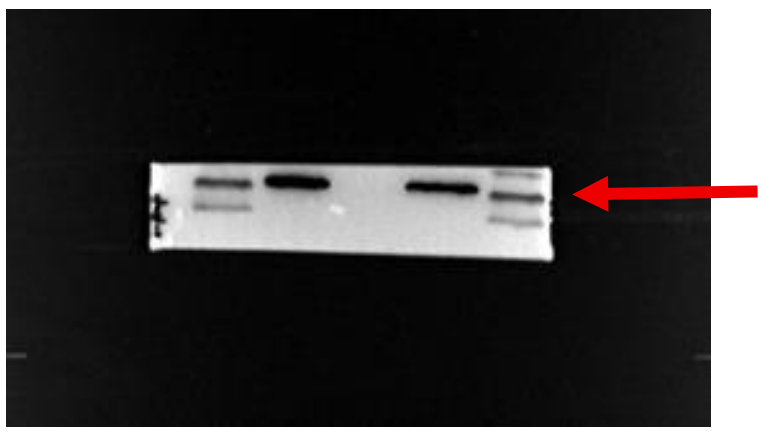

54KD

PDIA6

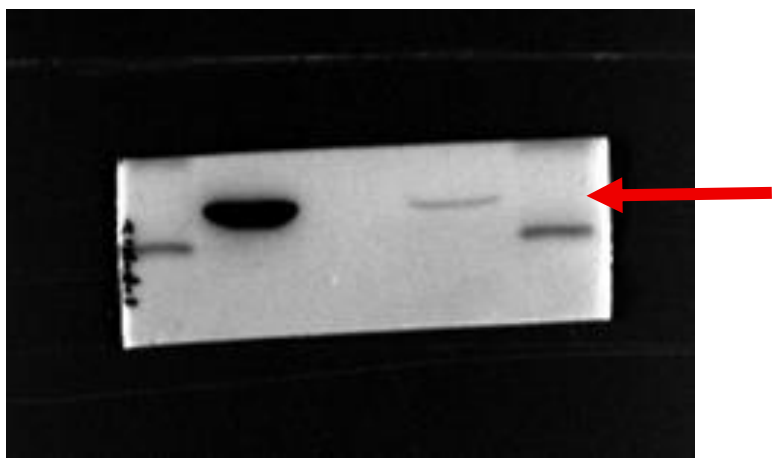

11KD

H4K12

IP: H4K12

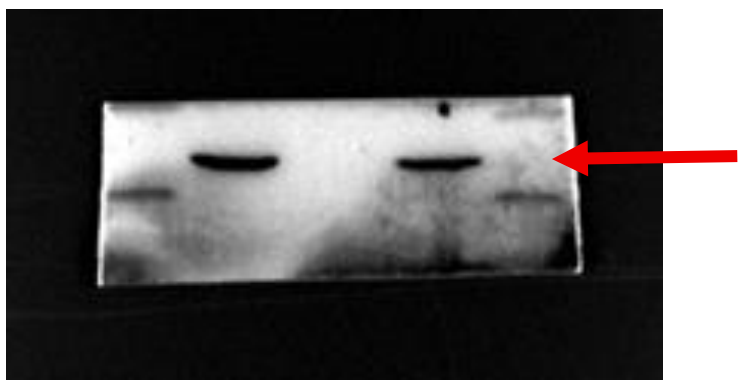

11KD

H4K12

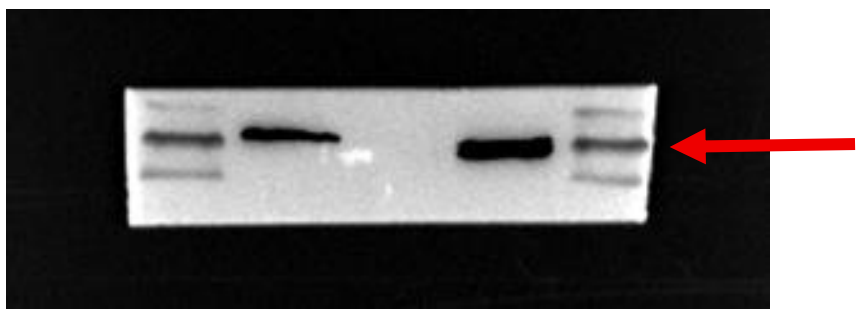

54KD

PDIA6

Fig. S5A

PDIA6 GAPDH

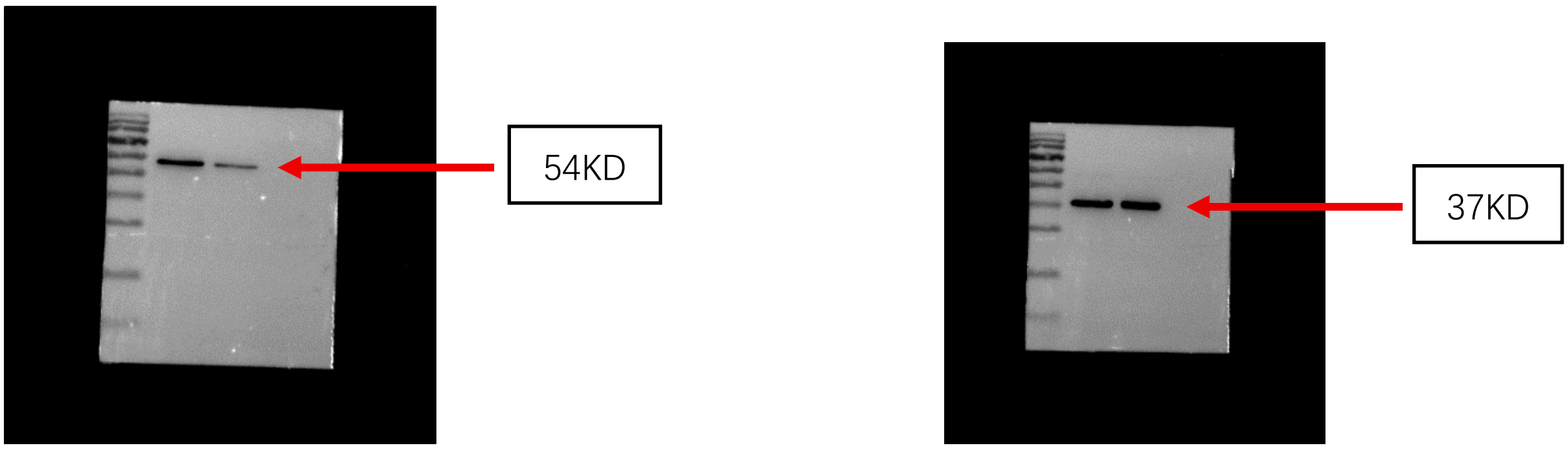

Fig. S5B

PDIA6 GAPDH

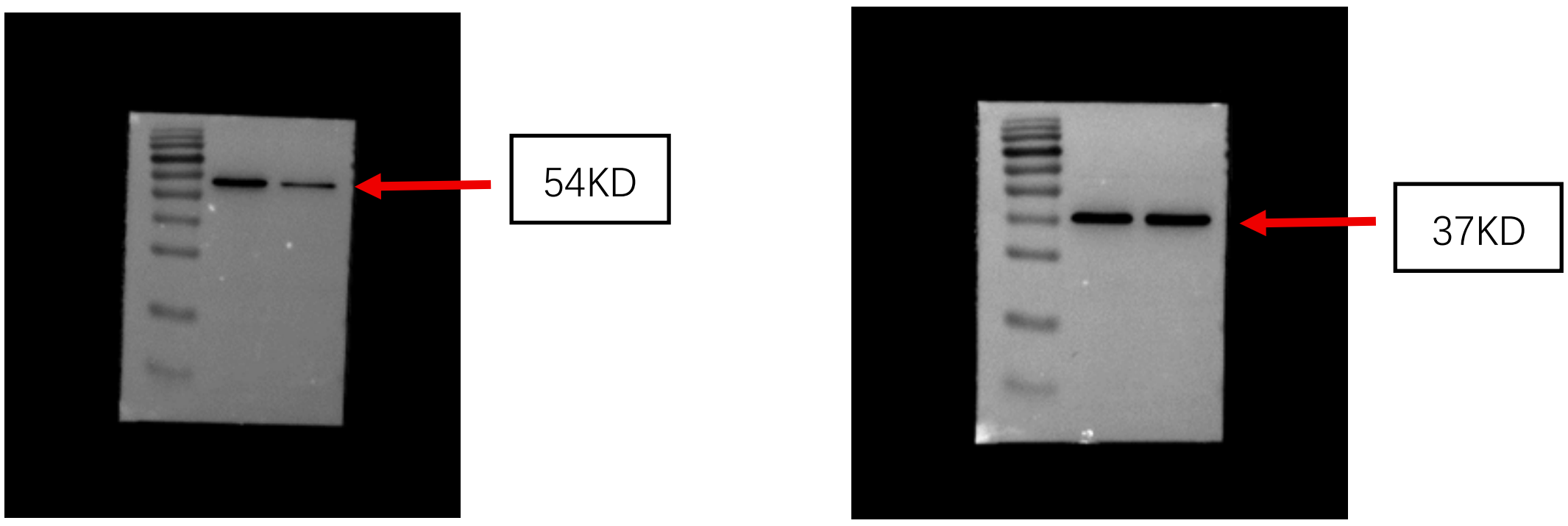

Supplement: Supplementary file 1 [file 41419_2026_8638_MOESM9_ESM.pdf]
