# Supplementary material for: Endoplasmic reticulum stress-induced histone lactylation mediating immune escape of gastric cancer via PDIA6 overexpression in dendritic cells
Source: Cell Death Dis. 2026 Mar 27;17(1):686. doi: 10.1038/s41419-026-08638-9 (PMC13444140; doi:10.1038/s41419-026-08638-9)
Supplement: Supplementary file 2 [file 41419_2026_8638_MOESM7_ESM.docx]

**Fig. S1 Construction of ERS model of gastric cancer cells and obtainment of BMDCs.**

(A, B) After TM stimulation of MFC, RNA and protein expression levels of GRP78 were detected. (C, D) After hypoxia stimulation, RNA and protein expression levels of GRP78 were detected. (E) ELISA was used to detect IL-12 secretion by iDC and mDC. (F) RT-qPCR was used to detect the RNA level of CD86, CD40, and MHCII. N = 3, data expressed as Mean ± SD for different groups. ** P* <0.05, *** P* <0.01

**Fig. S2 ERS promotes elevation of lactate level in gastric cancer cells.**

(A) After using a lactate detection kit to treat subcutaneous tumor tissue in mice, the supernatant was collected to detect the lactate content in tumor cells. (B) After TM/hypoxia stimulation of MFC, the supernatant was collected to detect lactate secretion level. (C) BGC-823 and AGS were co-cultured with TAMs and CAFs respectively, then the supernatant was collected to detect lactate secretion level. N = 3, data expressed as Mean ± SD for different groups. ** P* <0.05, *** P* <0.01

**Fig. S3 The detection of histone lactylation at H4K12 sites in DC**

(A) After BMDCs were treated with lactate, nuclear protein was extracted and the expression level of Pan-kla was detected by western blotting. (B) BMDCs histone protein was extracted. The modification was detected by mass spectrometry, and the secondary mass spectrometry peak showed lactylation of lysine at position 12. N = 3, data expressed as Mean ± SD for different groups. ** P* <0.05, *** P* <0.01

**Fig. S4 ERS enhanced the expression of PDIA6 via H4K12 lactylation in DC.**

(A) BMDCs were stimulated by gradient lactate, then RNA and protein levels of PDIA6 were detected. (B, C) Pretreated-supernatant and GSK were used to stimulate BMDCs, then RNA and protein levels of PDIA6 were detected. (D) GSK was treated with subcutaneous tumor mice in ERS status, then RNA level of PDIA6 was measured. (E) CO-IP experiment verified the relationship between H4K12 and PDIA6. (F, G, H) Lentivirus was used to infect BMDCs for H4K12 point mutation, then pretreated-supernatant/lactate were used to stimulate BMDCs, and ChIP-qPCR analysis of the indicated promoters was performed by using of H4K12 antibody. (I) GSK was treated with subcutaneous tumor mice in ERS status, and representative immunofluorescence staining was performed for H4K12 and PDIA6. N = 3, data expressed as Mean ± SD for different groups. ** P* <0.05, *** P* <0.01

**Fig. S5 ERS inhibits the immune function of DC by upregulating lactate level.**

(A, B) Supernatant was added to BMDCs after Si-PDIA6 treatment, and the expression of PDIA6 was detected by RT-qPCR and western blotting. (C, F) RT-qPCR was used to detect the RNA expression level of CD86, CD40, and MHCII in BMDCs. (D, G) The proportion of CD86 and MHCII was detected using flow cytometry. (E, H) BMDCs were co-cultured with CD8^+^ T cell, then the proportion of INF- γ and the secretion level of Granzyme B were measured. N = 3, data expressed as Mean ± SD for different groups. ** P* <0.05, *** P* <0.01

**Fig. S6 The role of ERS in gastric cancer microenvironment on the lactylation of H4K12 and PDIA6**

(A) Immunohistochemical was used to detect the expression of GRP78 in gastric cancer tissue. (B) The distribution of CD8^+^ T cell was detected according to GRP78 grouping. (C) Immunofluorescence double staining was used to detect the expression of H4K12 and PDIA6 in gastric cancer tissue. (D) Expression of GRP78 and PDIA6 in gastric cancer tissue was analyzed. (E) Overall survival curves for gastric cancer patients with different expression levels of PDIA6. N = 16, data expressed as Mean ± SD for different groups. * P <0.05，** P <0.01
