# Supplementary material for: Endoplasmic reticulum stress-induced histone lactylation mediating immune escape of gastric cancer via PDIA6 overexpression in dendritic cells
Source: Cell Death Dis. 2026 Mar 27;17(1):686. doi: 10.1038/s41419-026-08638-9 (PMC13444140; doi:10.1038/s41419-026-08638-9)
Supplement: Supplementary file 3 [file 41419_2026_8638_MOESM8_ESM.docx]

Fig. 2A

GRP78	GAPDH

37KD

78KD啊QD

Fig. 2B

GRP78	GAPDH

37KD

78KDDD

Fig. 5A

Pan-kla	GAPDH

37KD

MFC-CM

TM-MFC-CM

Fig. 5B

Pan-kla	GAPDH

37KD

Fig. 5C

Pan-kla	GAPDH

37KD

Fig. 5E

H4K12	Histone H3

15KD

11KD

Fig. 5F

H4K12	Histone H3

15KD

11KD

Fig. 5G

H4K12	Histone H3

15KD

11KD

Fig. 6C

PDIA6	GAPDH

37KD

54KD

Fig. 6D

PDIA6	GAPDH

37KD

54KD

Fig. 6I

PDIA6	GAPDH

37KD

54KD

Fig. 6J

PDIA6	GAPDH

54KD

54KD

Fig. 6K

PDIA6	GAPDH

54KD

54KD

Fig. S1B

GRP78	GAPDH

37KD

78KD

Fig. S1D

GRP78	GAPDH

78KD

78KD

Fig. S3A

Pan-kla	Histone H3

15KD

Fig. S4A

IP: PDIA6

Input

11KD

54KD

IP

IgG

PDIA6	H4K12

IP: H4K12

54KD

11KD

H4K12	PDIA6

Fig. S5A

PDIA6 GAPDH

37KD

54KD

Fig. S5B

PDIA6	GAPDH

37KD

54KD
